# Aberrant activation of epigenetic BRD9-DGAT1 axis promotes lipid droplets deposition and ferroptosis resistance in YAP-high prostate cancer

**DOI:** 10.1038/s41419-026-08746-6

**Published:** 2026-04-14

**Authors:** Xuejin Zhu, Zhimei Wen, Jinhai Wu, Sian Chen, Ran Xu, Bin Wang, Yingwen Zhu, Yanfei Chen

**Affiliations:** Department of Urology, Guangzhou Institute of Cancer Research, the Affiliated Cancer Hospital, Guangzhou Medical University, Guangzhou, China

**Keywords:** Cancer metabolism, Urological cancer, Cell death, Epigenetics

## Abstract

Aberrant interplay between epigenetics and metabolism contributes to prostate cancer (PCa) progression and represents a formidable challenge limiting the efficacy of drugs. Elucidation of the epigenetic underpinnings of prostate cancer (PCa) could provide promising insights into the drivers of therapy resistance. Through an unbiased siRNA screen of mSWI/SNF family members, which play a significant role in tumorigenesis, we identified Bromodomain containing 9 (BRD9) as an essential gene for PCa growth. Targeting BRD9 abolished PCa colony formation and migration in vitro, and inhibited orthotopic tumor growth in vivo. YAP/TEAD4 complex bound to the BRD9 promoter to elevate its levels. Integrated CUT&Tag-seq and RNA-seq analyses revealed DGAT1 as an important BRD9 effector. Mechanistically, BRD9 interacted with SREBP1 to co-occupy the DGAT1 promoter, increasing the H3K4me3 enrichment and chromatin accessibility. Additionally, the YAP-BRD9 axis enhanced the lipid droplets (LDs) formation, ferroptosis resistance, and tumorigenesis via inducing DGAT1. The pharmacological inhibition (or depletion) of BRD9 suppressed LDs formation, restored ferroptosis sensitivity, and PCa malignancy. Overall, the BRD9-SREBP1-DGAT1 axis represents a potential epigenetic therapeutic target for YAP-high PCa.

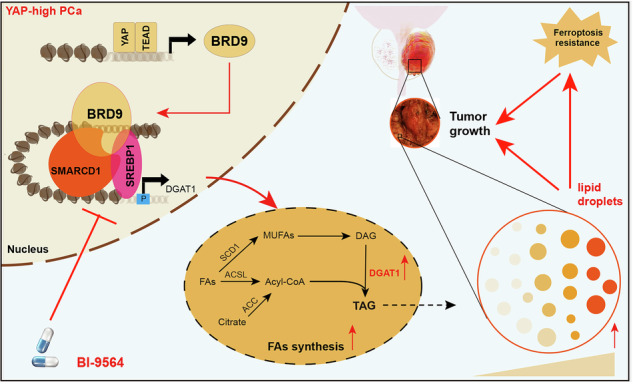

## Introduction

As one of the most prevalent malignancies among men worldwide, prostate cancer (PCa) caused nearly 400 thousand deaths annually [1]. The majority of localized PCa with early stages could be cured by surgical prostatectomy, whereas the treatment efficacy for advanced cases was largely abrogated by the acquired resistance [2]. Traditional endocrine therapies, including androgen deprivation therapy (ADT) and antiandrogen therapy, are the main strategy for advanced PCa. However, a substantial of these cases would gradually develop into castration-resistant PCa (CRPC) stage due to reactivation of androgen receptor (AR) or other AR-independent signaling [3]. Besides, immunotherapy is suitable for PCa patients with advanced disease or those resistant to ADT, including immune checkpoint inhibitors, CAR-T cell therapy, or therapeutic vaccines, etc [4–6]. However, there is less T cell infiltration in the PCa microenvironment, leading to insufficient immune activity [7]. Therefore, in-depth depiction of underlying molecular mechanisms related with PCa tumorigenesis is critical to the investigation of novel targets and therapies.

As reported, epigenetic reprogramming has emerged as a vital underlying mechanism leading to the initiation, progression, and drug resistance of PCa. Epigenetics includes histone or ‌non-histone‌ modifications, like DNA methylation or chromatin remodeling, and those do not change the DNA sequences [8]. Known as the BRG1/BRM-associated factor (BAF) complexes, the SWI/SNF family of chromatin-remodelling complexes belong to essential factors of nucleosome positioning. Mammalian SWI/SNF (mSWI/SNF) complexes contain three subfamilies: canonical BAF (cBAF), polybromo-associated BAF (PBAF), and the GLTSCR1 or GLTSCR1L-containing and BRD9-containing (GBAF) complex, which is also named as the non-canonical BAF (ncBAF) [9, 10]. Emerging evidence suggested that the frequent involvement of altered mSWI/SNF complexes in both human cancer and neurological disorders, suggesting new mechanisms and accompanying routes toward therapeutic intervention [11]. For instance, SWI/SNF ATPase degradation is effective to treat enhancer-binding transcription factor-addicted cancers, like AR–FOXA1-driven PCa, via disrupting enhancer-linked oncogenic gene machinery [12]. SWI/SNF is a multi-subunit chromatin-remodelling complex that uses energy from ATP hydrolysis to reposition or eject nucleosomes at non-coding regulatory elements, thereby enabling free DNA access for the transcriptional machinery. Members of the SWI/SNF family are closely associated with drug resistance and malignant progression in prostate cancer. For instance, Bengul Gokbayrak et al. identified and validated SMARCC2, a core subunit of SWI/SNF complex (also known as BAF complex), and DPF2, a canonical BAF complex specific subunit, that was selectively essential and elevated in enzalutamide-resistant prostate cancer models [13]. Phillip Thienger also discovered that SWI/SNF ATPase SMARCA4 depletion interfered with the master transcriptional regulator TCF7L2 (TCF4) in WNT-signaling dependent AR-negative CRPC (CRPC-WNT) [14]. Of note, the bromodomain-containing protein 9 (BRD9), as a component of the newly identified SWI/SNF complex, which has the potential to be a diagnostic and prognostic biomarker in PCa [15]. Bromodomain-containing proteins recognize acetylated lysine residues on histones and epigenetically regulate transcription, chromatin remodeling, and histone modification. BRD9 blockade could modulate AR-targets and metabolic pathways, indicating the possibility that BRD9 may serve as a treatment target in therapy-resistant PCa [16]. Besides, BRD9 interacts with androgen receptor (AR) and CTCF to modulate the AR-dependent gene expression, uncovering a novel druggable target for AR-positive PCa [17]. Whether BRD9 could modulate other AR-independent signaling pathways is still unknown. In addition, we still do not know whether other SWI/SNF family members are involved in the malignant progression of PCa.

Increasing studies have elucidated that lipid metabolism is aberrantly reprogrammed in malignancies, especially in PCa. As reported, lipogenesis process is enhanced to fulfill the necessity of required membrane biogenesis [18]. Of note, lipid droplets (LDs) are dynamic cellular organelles required for depositing neutral lipids such as triacylglycerols and cholesteryl esters, with a phospholipid monolayer membrane and diverse associated proteins [19]. LDs participate in lipid synthesis, catabolism, and transport processes by interacting with other organelles, and they play essential roles in disease pathogenesis, and biomedical applications like drug delivery and cancer treatment [20]. For instance, Ying Meng et al. proved that PFKL, one glycolytic enzyme, governs lipolysis by enhancing LDs-mitochondria tethering to enhance β-oxidation in liver cancer [21]. Besides, HIF-2α/LINC02609/APOL1-mediated lipid storage promotes endoplasmic reticulum homeostasis and regulates tumor progression in clear-cell renal cell carcinoma [22]. There is also evidence that LDs play an important role in the progression of PCa. Lijie Zhou et al. reported that ACSS3 inhibits PCa progression through inhibiting lipid droplet-associated protein PLIN3 [23]. Moreover, PIM1 could potentiate LDs accumulation to promote proliferation and survival in PCa [24]. As well documented, sterol regulatory element-binding proteins (SREBPs) are important transcription factors that sustain the transcriptome involved in lipid storage or utilization, and play a vital role in lipid metabolism under both physiological or pathological situations. For instance, EXO1/P53/SREBP1 axis-regulated lipid metabolism promotes PCa progression [25]. Oncogenic activation of PI3K-AKT-mTOR signaling could further inhibit ferroptosis via SREBP-mediated lipogenesis in PCa [26]. Nevertheless, the intrinsic epigenetic regulatory mechanism of SREBPs in regulating LDs remains indefinite.

In this study, we identified that novel SWI/SNF subunit BRD9 could enhance PCa progression and LDs formation via AR-independent manner. We uncovered the in-depth epigenetic mechanisms of BRD9-SREBP1 axis in PCa LDs. Importantly, combing BRD9 inhibition with ferroptosis induction elicited a significant tumor regression in xenograft mouse models for PCa with hyperactivation of YAP signaling.

## Results

### Identification of BRD9 as a novel hazard mSWI/SNF subunit associated with PCa

**Fig. 1:**
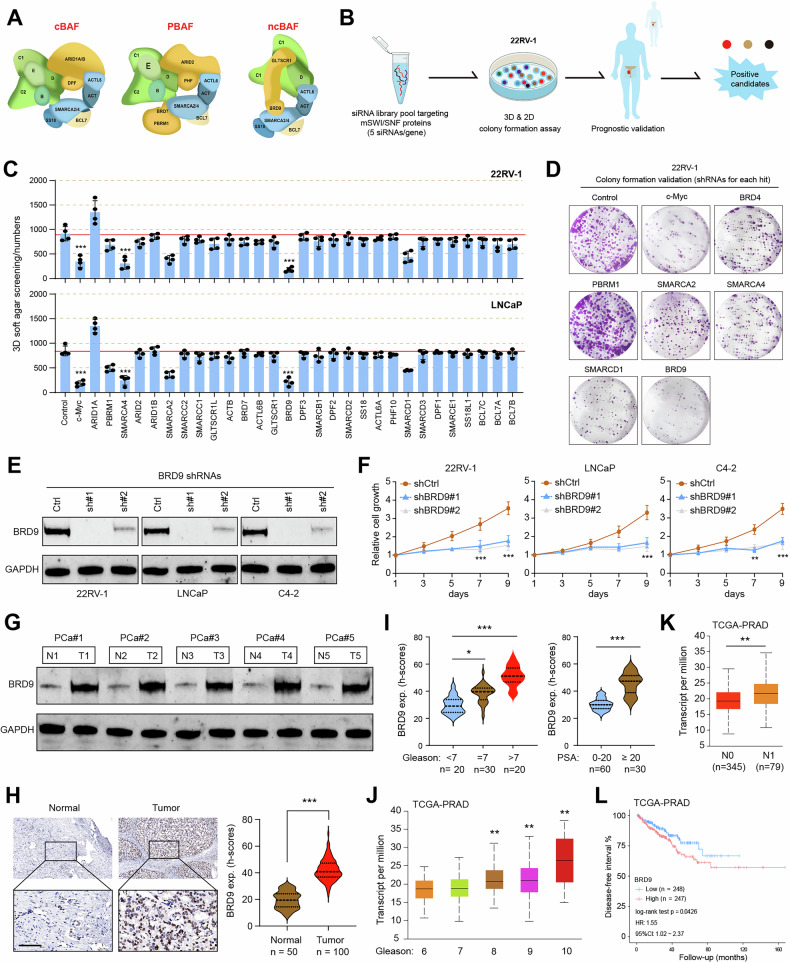
Screening and validation of BRD9 as a novel hazard mSWI/SNF subunit in PCa. **A** Illustration of canonical BAF (cBAF), polybromo-associated BAF (PBAF), and noncanonical BAF (ncBAF) subunit composition. **B** Schematic diagram of the screening strategy of this study. 22RV-1 cells were transfected with siRNA pools targeting mSWI/SNF subunits for 48 h, siRNAs targeting c-Myc as positive control, or non-targeting siRNA as the negative control, cells were then applied for 3D soft agar colony formation assay. **C** Soft agar colony quantification for the screening. The c-Myc was used as the the positive control screening. The screening experiment were performed by four biological replicates. **D** Representative colony formation graphs of positive/negative hits or controls transfected with specific shRNAs in 22RV-1 cells, individually. **E** Western blot assays showing the knockdown efficacy of BRD9 in PCa cells. **F** CCK-8 experiments showing the cell growth rates in PCa cells with or without BRD9 knockdown. **G** Western blot assays showing the BRD9 protein levels in normal and paired PCa tissues. **H** BRD9 immunohistochemistry (IHC) staining of representative normal or tumor tissues. Scale bar = 50 μM. Quantification data was shown on the right. **I** Quantification of BRD9 h-scores in PCa-dataset and correlation with Gleason scores or PSA levels. Correlation analysis of BRD9 levels with Gleason scores (**J**), and lymph node metastasis (**K**) in TCGA-PRAD patients. **(L)** Kaplan–Meier (K-M) survival curve analysis was conducted in BRD9-high and -low patients to show the prognostic difference. *P < 0.05, **P < 0.01, ***P < 0.001, and ns means no significance. Error bars represent SEM, two-tailed Student’s *t* test.

In order to screen the novel mSWI/SNF subunits that are required for PCa growth, we conducted an siRNA screen based on the ON-TARGETplus siRNA library of all human mSWI/SNF subunits [27] (Fig. 1A, B). The screening method was mainly performed by the 3D anchorage-independent growth. Meanwhile, the non-targeting sequences were used as the negative control and a well-known oncogene, c-Myc, was used as a positive control. During the initial siRNA screening process in 22RV-1 or LNCaP cells, we identified ~5 subunits that may alter 3D growth upon depletion (Fig. 1C). In order to explore candidates and exclude the false positives, we designed the specific shRNAs that target the 5 subunits and repeated the colony formation assays. Then, we were able to narrow down the range and obtain the following 3 hits: SMARCA2, SMARCA4, and BRD9 (Fig. 1D). Among them, SMARCA2/4 was reported to mediate the enhancer accessibility and targeting SWI/SNF ATPase induces potent inhibition of PCa growth [9]. Compared with SMARCA2/4, BRD9 was less reported in PCa. Therefore, we were interested in BRD9, as it may also modulate PCa progression. To confirm this speculation, we transfected 3 PCa cell lines (22RV-1, LNCaP, and C4-2) with two individual BRD9 shRNAs and determined the effects on cell proliferation (Fig. 1E). Indeed, BRD9 knockdown could notably reduce cell proliferation, as revealed by MTS proliferation assays (Fig. 1F). We also obtained 5 paired PCa samples via the laparoscopic resection technique and detected that BRD9 expressions were higher in tumors than those in normal tissues (Fig. 1G). To further evaluate the clinical relevance of BRD9 in PCa, we conducted the IHC with an antibody against BRD9 in a PCa tissue microassay (TMA) containing 150 specimens. IHC analysis of these specimens revealed higher BRD9 expressions in tumors than in normal tissues (Fig. 1H). Besides, the BRD9 IHC h-scores were positively associated with the Gleason scores and PSA levels (Fig. 1I). We also obtained the expression matrix of 497 PCa samples from the TCGA-PRAD cohort and confirmed the positive relationships between BRD9 expressions and Gleason scores (Figure IJ). Patients with lymph node metastasis (N1) tended to have higher BRD9 expressions than those without lymph node metastasis (N0) (Fig. 1K). Kaplan–Meier survival analysis revealed that BRD9 proteins were positively associated with worse prognosis in tumors with low BRD9 expression (*P* = 0.0426; Fig. 1L). Taken together, these data implicated a causal role of BRD9 in prostate tumorigenesis, and we focused on this risk mSWI/SNF subunit for further in-depth research.

### BRD9 depends on Bromodomain (BRD) to potentiate PCa malignancy in vitro and in vivo

**Fig. 2:**
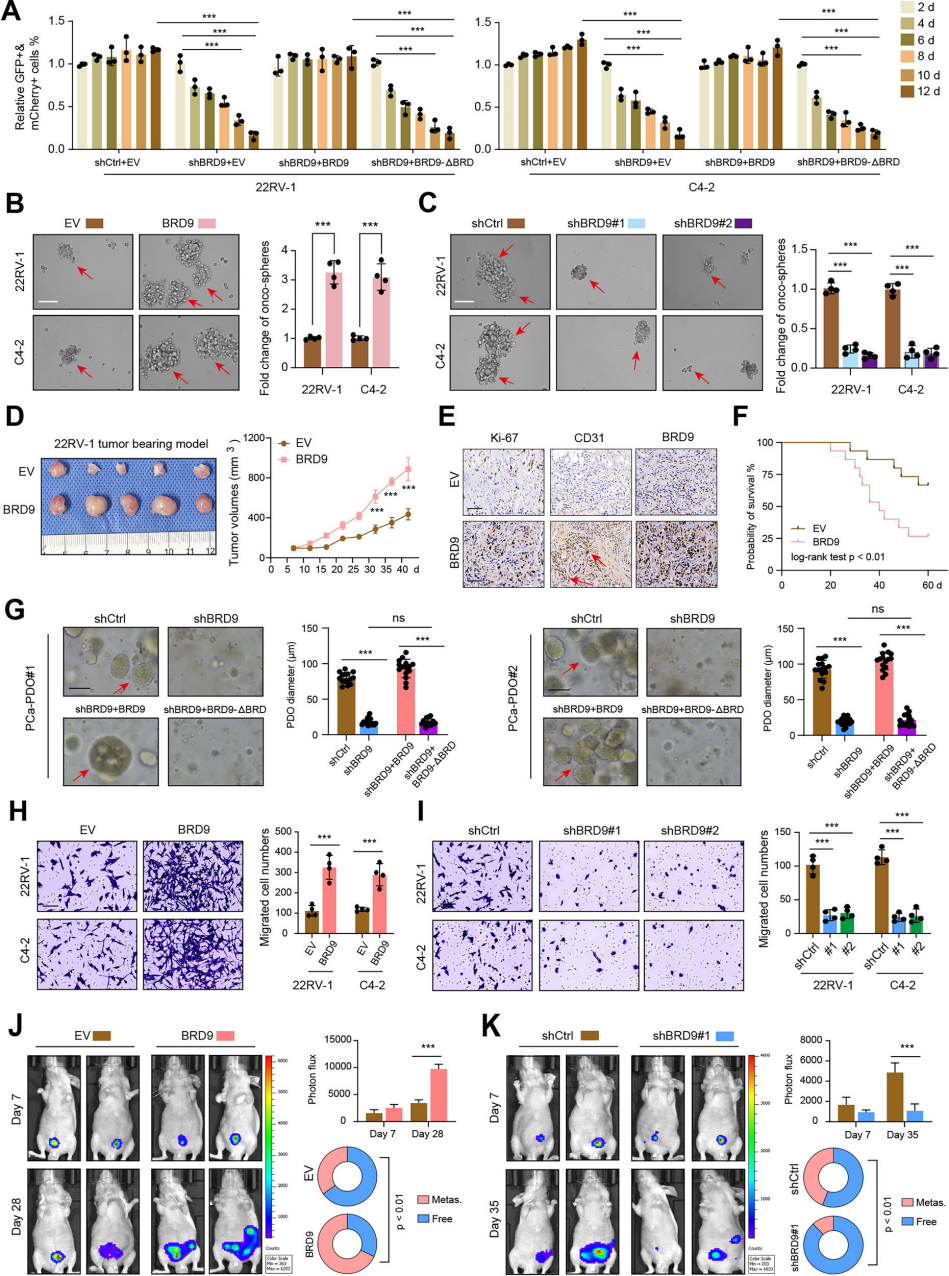
BRD9 is required for malignant growth and metastasis of PCa. **A** Competition-based assay to confirm the effect of BRD9-KD on proliferation of 22RV-1 and C4-2 cells. BRD9-WT or -ΔBRD (linked to GFP, MSCV-based vector) was over-expressed in 22RV-1 and C4-2 cells before the expression of BRD9 shRNAs (linked to mCherry, LMN vector). The percentage of double positive cells were quantified and normalized to day 2 values (n = 4). EV means the empty vector control. **B** Representative graphs of tumor spheres in PCa cells with or without BRD9 overexpression. **C** Representative graphs of tumor spheres in PCa cells with or without BRD9 deficiency via shRNAs-mediated knockdown. **D** Representative tumor graph (left) showing the results of 22RV-1 tumor bearing model. The quantification data of tumor volumes from EV and BRD9 groups was shown on the right. **E** The representative pictures showing the Ki-67/CD31 IHC staining results in tumors from EV or BRD9 group. **F** Kaplan–Meier analysis showing the survival differences in mice from EV and BRD9 group. **G** Representative PDOs graphs and statistical analysis in BRD9-KD PDOs with BRD9-WT or BRD9-ΔBRD restoration. **H** Representative transwell assays in PCa cells with or without BRD9 overexpression. The quantification data was exhibited on the right. **I** Transwell assays (left) and quantification analysis (right) were conducted in control or BRD9-KD PCa cells. **J** PCa orthotopic implantation model showing the growth and metastasis of PCa cells with or without BRD9 overexpression. The BIL and metastatic rates were shown on the right. **K** PCa orthotopic implantation assays and relevant quantification analysis in mice from shCtrl and shBRD9 groups. *P < 0.05, **P < 0.01, ***P < 0.001, and ns means no significance. Error bars represent SEM, two-tailed Student’s *t* test.

To further confirm the functional role of BRD9 in PCa, we generated the stable BRD9-overexpressing 22RV-1 or C4-2 cells (Figure S1A). The EdU cell proliferation assays suggested that BRD9 potentiated the DNA synthesis process in PCa cells (Figure S1B). The competitive proliferation experiments were used to assess the effect of BRD9 depletion on the growth of PCa cells. Expectedly, 22RV-1 and C4-2 cells were significantly outcompeted by nontransduced cells during culturing (Fig. 2A). However, the over-expression of a human wild-type BRD9 cDNA not recognized by BRD9 shRNA could completely rescue the impaired cell growth caused by shRNA (Fig. 2A). Interestingly, the human truncated BRD9-ΔBRD cDNA failed to reverse the impeded cell growth (Fig. 2A). Next, we conducted the sphere-formation assay and found that BRD9 overexpression in 22RV-1 and C4-2 could notably enhance the formation of spheres and self-renewal capacity (Fig. 2B). In contrast, BRD9 inhibition via transfection of shRNAs could suppress the self-renewal efficacy and tumorspheres (Fig. 2C). Then, we utilized the control or BRD9-overexpressing 22RV-1 cells to construct the subcutaneous tumor model. As evidenced by the tumor growth curve, tumor weight, and IHC results, we observed enhanced tumor growth, angiogenesis, and progression in vivo (Fig. 2D, E, and Figure S1C). Notably, BRD9 overexpression also shortened the survival months of mice (Fig. 2F). In contrast, tumors with BRD9 knockdown showed lower growth rates compared with those derived from control cells (Figure S1D). Furthermore, we obtained fresh PCa resected samples and constructed the organoid models from two patients to determine the clinical meaning of BRD9. We observed that shRNAs-mediated BRD9 knockdown could obstruct the growth of organoids, which could be completely rescued by BRD9 overexpression (Fig. 2G). Consistently, BRD9-deficient PDOs failed to restore the growth capacity with restoration of BRD9-ΔBRD (Fig. 2G). To further explore the functional roles of BRD9 in PCa metastasis, we conducted the migration assays in vitro. The results revealed that BRD9 overexpression enhanced the migration of 22RV-1 and C4-2 cells (Fig. 2H). However, BRD9 knockdown inhibited the migration phenotypes of these cells, compared with control cells (Fig. 2I). The wound-healing assays also confirmed that BRD9 could enhance the rates of migration, whereas BRD9 inhibition hindered the migration (Figure S1E). Last of all, we also intended to determine the role of BRD9 in orthotopic PCa models. Briefly, we generated the murine subcutaneous tumor model using PC3-luciferase cells with or without BRD9 overexpression. Then, we trimmed the tumors to the appropriate size and inserted these tissues into the murine prostate section. As indicated by the bioluminescence (BIL) imaging, BRD9 overexpression could significantly enhance PCa growth and distant metastasis (Fig. 2J). In contrast, PC3 cells with BRD9 deficiency remarkably impeded PCa growth and lung metastasis, as evidenced by BIL signals (Fig. 2K). Collectively, these results indicated that BRD9 is an essential oncogene for PCa growth and metastasis.

### YAP/TEAD complex upregulates BRD9 expression in PCa

**Fig. 3:**
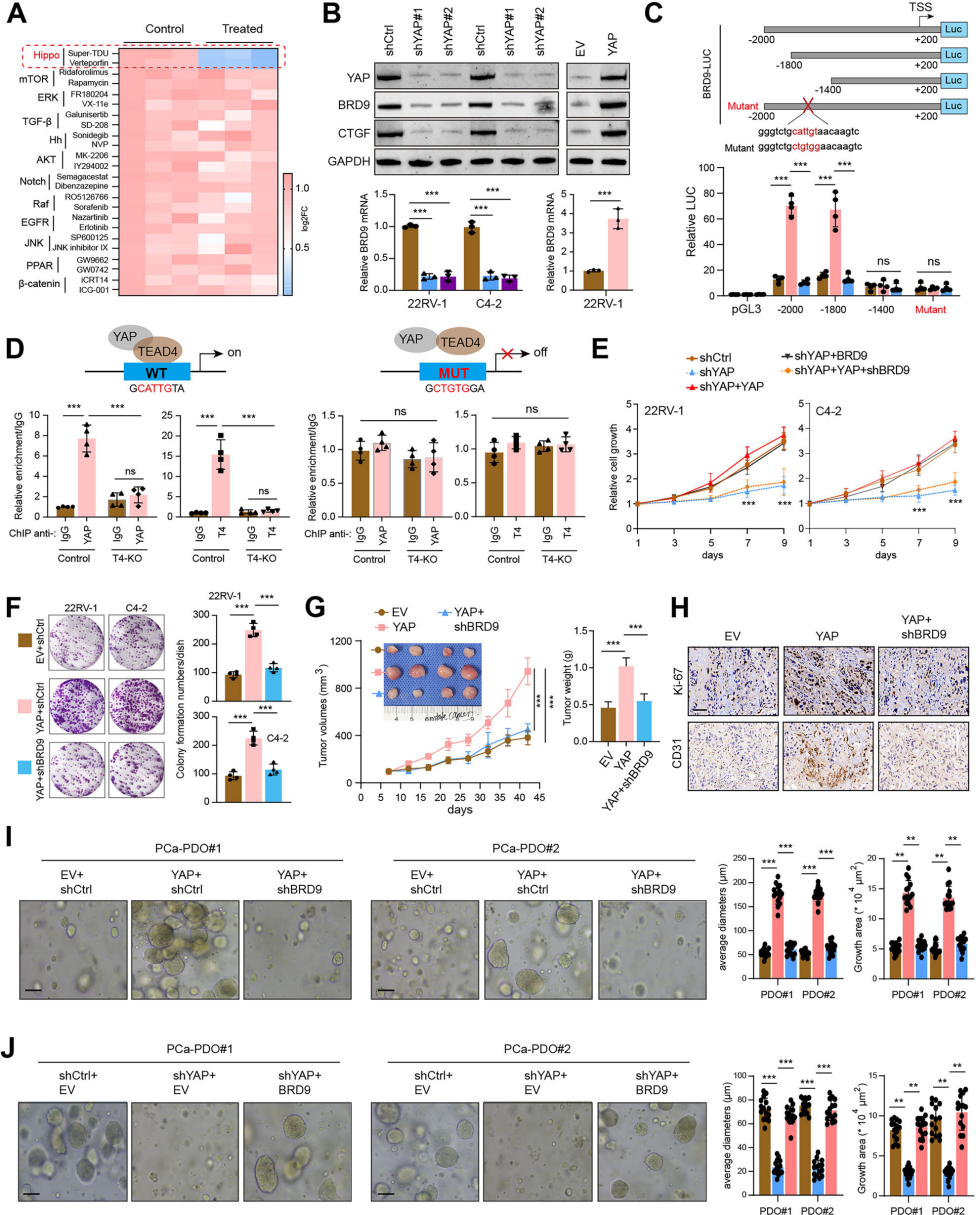
YAP/TEAD4 complex upregulates BRD9 levels and promotes PCa malignancy. **A** Heatmap revealing the mRNA levels of BRD9 in 22RV-1 cells treated with the indicated compounds and control vehicle treatment. **B** Western blot and RT-qPCR assays showing the protein or mRNA levels in control or YAP-KD PCa cells. The YAP-downstream target, CTGF, is used as the positive control. **C** The BRD9 promoter-driven luciferase reporters and luciferase activity in 22RV-1 cells (*n* = 4). The TEAD4-binding element was highlighted and mutated. **D** ChIP-qPCR assays showing the binding ability of YAP/TEAD4 on the promoter region of BRD9 (left) in 22RV-1 cells with or without TEAD4-KO. The BRD9-promoter was accordingly mutated, and the ChIP-qPCR assays were repeated on the right. **E** CCK-8 assays showing the cell proliferation abilities in YAP-depleted PCa cells with or without BRD9 restoration. **F** Representative colony formation pictures and quantification data revealing the colony growth abilities in YAP-sustained PCa cells with or without BRD9 inhibition. **G** Representative tumor pictures (upper) and quantification data (lower) exhibiting the in vivo growth of tumors derived from YAP-sustained PCa cells with or without BRD9 inhibition. **H** Representative IHC graphs showing the CD31/Ki-67 levels in tumors derived from mice in (**G**). **I** Representative PDOs graphs and quantification statistical analysis in YAP-activated PDOs with or without BRD9 inhibition. **J** Representative PDOs graphs and quantification statistical data in YAP-KD PDOs with or without BRD9 over-expression. *P < 0.05, **P < 0.01, ***P < 0.001, and ns means no significance. Error bars represent SEM, two-tailed Student’s *t* test.

We thus wondered about the upstream signals that govern BRD9 expression to drive PCa tumorigenesis. To figure out the potential mechanisms, we performed a screen in 22RV-1 cells via a panel of compounds that inhibit several well-known oncogenic signals in PCa, including the YAP, AKT-mTOR, Hedgehog, and Notch, among others. Surprisingly, we observed more than 50% reductions in BRD9 mRNA levels when the YAP signal was blocked via verteporfin or Super-TDU treatment in 22RV-1 cells (Fig. 3A). We also confirmed that BRD9 levels were down-regulated in YAP-KD cells, and enforced overexpression of YAP could activate the BRD9 expression (Fig. 3B). Then, we generated a series of luciferase reporters driven by various fragments of the BRD9 promoter and identified one TEAD-binding element that was responsible for the stimulatory effects of YAP (Fig. 3C). ChIP-qPCR assays further validated that YAP could bind to the promoter region of BRD9 in a TEAD4-dependent manner, which could be abrogated by the above-constructed BRD9-mutant (Fig. 3D). We also queried the TCGA-PRAD dataset and confirmed the positive relationships between YAP1 and BRD9 levels (Figure S2A). Consistently, we also observed this relationship in other malignancies, like TCGA-KIRC, TCGA-BRCA, or TCGA-LIHC (Figure S2A). A positive correlation between YAP1 and BRD9 proteins was further found in TMAs consisting of 120 PCa samples (*P* < 0.0001; Figure S2B). Thus, these findings suggested that YAP bound to the BRD9 promoter and stimulated its expression in a TEAD4-dependent manner. Then, we intended to confirm the functional relationships between YAP and BRD9. Noticeably, ectopic overexpression of BRD9 could completely rescue the growth inhibition in 22RV-1 and C4-2 cells with YAP deficiency, whereas BRD9-KD could largely inhibit the effects of reconstituting YAP in YAP-deficient cells (Fig. 3E). In addition, YAP overexpression could enhance colony formation, self-renewal, and migration abilities of PCa cells, which could be inhibited by BRD9 inhibition (Fig. 3F and Figure S2C). Expectedly, knockdown of BRD9 also markedly abrogated the YAP-driven PCa growth and angiogenesis in vivo, as implicated by tumor growth curve, tumor weight, IHC analysis, and prognostic survival analysis (Fig. 3G, H, and Figure S2D). Based on the established PCa organoids in Fig. 2F, we further demonstrated that BRD9 knockdown could remarkably suppress YAP-induced PCa organoids growth (Fig. 3I-J). Collectively, our data indicated that YAP could potentiate PCa malignant progression via upregulating BRD9 expression.

### BRD9 modulates fatty acid metabolism and occupies the DGAT1 promoter in PCa

**Fig. 4:**
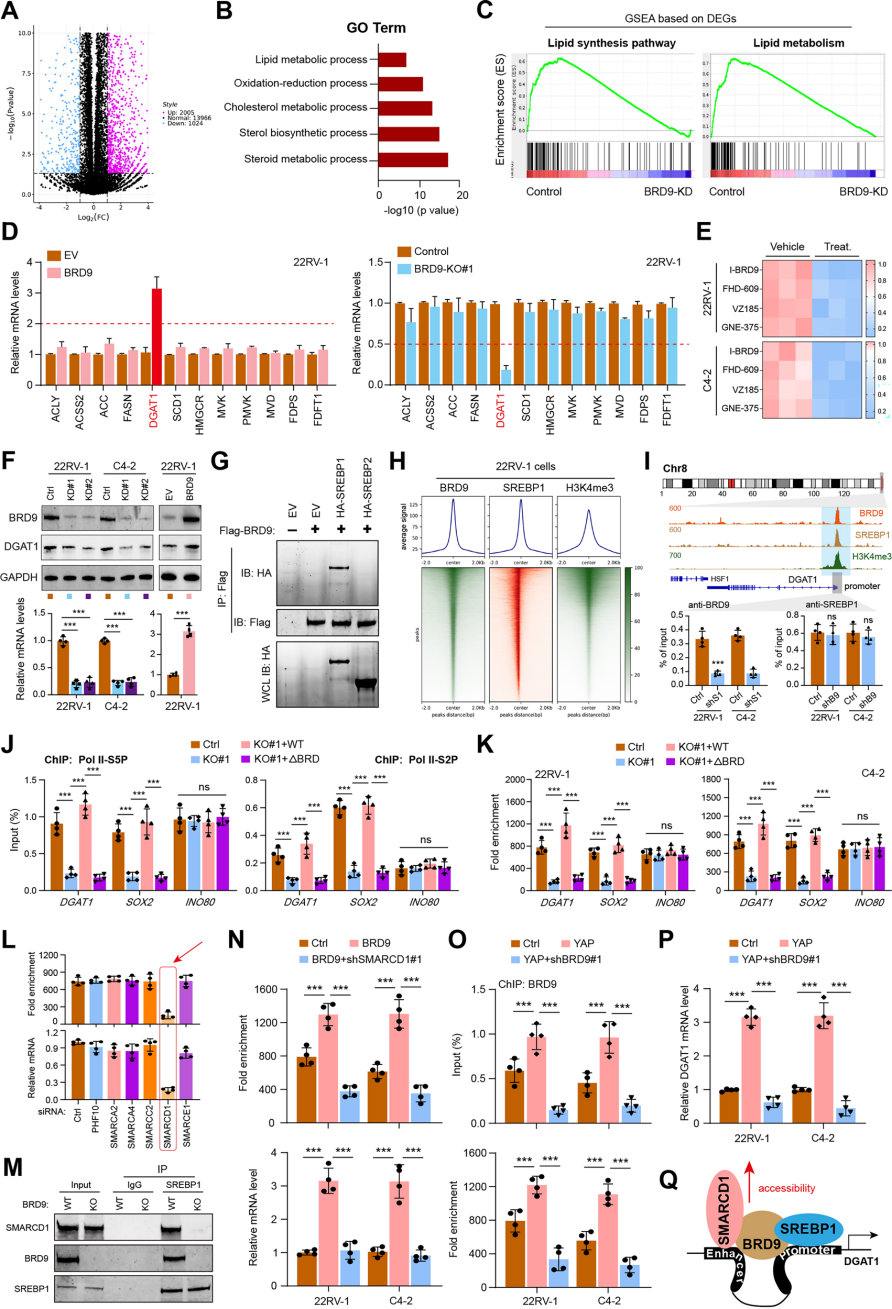
BRD9 couples with SREBP1 to activate DGAT1 levels in PCa. **A** The volcano plot was generated to show differentially expressed genes (DEGs) in 22RV-1 cells with or without BRD9 depletion. Purple showed the up-regulated genes, whereas blue represented the decreased genes. **B** Bioinformatic pathway analysis of all the DEGs in BRD9-deleted 22RV-1 cells versus control cells. **C** GSEA results were shown to reveal the relationships between BRD9 and lipid metabolism. **D** The RT-qPCR analysis showing the mRNA alterations of genes involved in lipid metabolism in BRD9-overexpressing (left) or BRD9-KO (right) 22RV-1 cells versus control cells. **E** Heatmap revealing the mRNA levels of DGAT1 in 22RV-1 cells treated with the BRD9 inhibitors and control vehicle. **F** Western blotting (upper) and RT-qPCR analysis (below) detecting the DGAT1 expressions in BRD9-KD or BRD9-overexpressing PCa cells versus control cells. **G** Co-IP assays detecting the interaction between BRD9 and SREBP1/2 in 293 T cells transfected with indicated plasmids. **H** CUT&Tag-sequencing and peak heatmap showing the co-binding pattern of BRD9/SREBP1/H3K4me3 in indicated regions. **I** IGV tracks showing the binding peaks of BRD9/SREBP1/H3K4me3 at the DGAT1 promoter region (upper). ChIP-qPCR assays showing the BRD9 or SREBP1 binding ability at the DGAT1 promoter with specific BRD9 or SREBP1 knockdown, individually (lower). **J** ChIP-qPCR analysis of Pol II-S5P (left) and S2P (right) in the promoter regions of the indicated genes in Ctrl and BRD9-KO 22RV-1 cells stably restoring WT BRD9 or its BRD9-Δbromodomain mutant as indicated. The INO80 gene was used as the negative control. **K** ChIP assays showing the altered chromatin accessibility in indicated regions in Ctrl and BRD9-KO 22RV-1 cells stably restoring WT BRD9 or its BRD9-Δbromodomain mutant. **L** ChIP assays (upper) and corresponding RT-qPCR assays (below) to screen potential chromatin co-activators that govern DGAT1. **M** Western blot analysis revealing the SREBP1-SMARCD1 interactions in Ctrl and BRD9-KO 22RV-1 cells. **N** ChIP assays (upper) and corresponding RT-qPCR assays (below) showing the chromatin accessibility and mRNA levels of DGAT1 in BRD9-overexpressing cells with or without SMARCD1 inhibition. **O** ChIP assays and RT-qPCR assays (**P**) showing the alterations of BRD9-binding ability, chromatin accessibility, and mRNA levels of DGAT1 in YAP-overexpressing cells with or without BRD9-KD. **Q** Illustration of BRD9/SREBP1-mediated epigenetic remodeling facilitating SMARCD1 recruitment. *P < 0.05, **P < 0.01, ***P < 0.001, and ns means no significance. Error bars represent SEM, two-tailed Student’s *t* test.

To further investigate the underlying molecular mechanism in BRD9-mediated PCa tumor progression, we performed RNA sequencing (RNA-seq) in control and BRD9-depleted 22RV-1 cells. The 3029 differentially expressed genes (DEGs) were detected when BRD9 was down-regulated, as shown by volcano plot (Fig. 4A). Gene Ontology and pathway analyses suggested that gene sets involved in lipid metabolic processes were most significantly highlighted (Fig. 4B). We performed an unbiased Gene Set Enrichment Analysis (GSEA) of these published datasets to confirm the consistent finding (Fig. 4C). To further investigate the mechanisms by which BRD9 modulates lipid metabolism, we detected the transcription of a series of lipid metabolism-related signature in BRD9-overexpressing and BRD9-KO cells. Compared with other genes, the expression levels of DGAT1 were increased (fold change > 3) in BRD9-overexpression cells, but were consistently inhibited (fold change < 0.5) by BRD9-KO in 22RV-1 cells (Fig. 4D). We further utilized the selective BRD9 antagonists, like I-BRD9, FHD-609, VZ185, or GNE-375, to treat PCa cells. Consistently, these BRD9 antagonists could significantly inhibit DGAT1 mRNA levels (Fig. 4E). We further verified that BRD9-KD could downregulate DGAT1 levels and BRD9 overexpression could elevate its expression in PCa cells (Fig. 4F). The positive relationships between BRD9 and DGAT1 expressions were demonstrated in TCGA-PRAD, TCGA-HNSCC, or TCGA-PAAD datasets (Figure S3A). RT-qPCR analysis also demonstrated that YAP could increase DGAT1 levels, whereas BRD9 overexpression could restore the DGAT1 levels in YAP-deficient cells (Figure S3B). In addition, AR knockdown failed to inhibit the DGAT1 levels in BRD9-overexpressing PCa cells, indicating the AR involvement was excluded in the context of the BRD9/DGAT1 axis (Figure S3C). Sterol regulatory element binding proteins (SREBPs) belong to a transcriptional regulator that controls the expression of genes for fatty acid and cholesterol biosynthesis. Co-IP assays revealed that BRD9 proteins were associated with SREBP1, but not with SREBP2 (Fig. 4G). Intriguingly, the bromodomain (147-228 aa) domain within BRD9 was responsible for the endogenous interactions between BRD9 and SREBP1 (Figure S3D). In addition, the BRD9-Δbromodomain truncation mutant failed to increase DGAT1 levels and cholesterol biosynthesis, implicating the essential role of the bromodomain (Figure S3D-E). We also adopted Proximity Ligation Assays (PLA) and immunofluorescence stainings (IF), which further showed that BRD9 and SREBP1 were largely colocalized in the nuclei and cytoplasm across different PCa cells (Figure S3F).

We therefore questioned whether BRD9 complexes with SREBP1 to regulate DGAT1 levels. To test this hypothesis, we performed the CUT&Tag-sequencing with BRD9 and SREBP-1 antibodies in 22RV-1 cells. We used a pie chart to display the distribution of chromatin regions bound by BRD9 and confirmed that BRD9 mainly binds to promoters (Figure S3G). Our CUT&Tag-seq data revealed that BRD9 overlapped primarily with H3K4me3, along with SREBP1 (Fig. 4H). A closer examination of BRD9 and SREBP1 peaks at DGAT1 gene loci revealed that BRD9 and SREBP1 were preferentially recruited in the promoter regions, marked by H3K4me3 modifications (Fig. 4I). Then, we questioned whether BRD9 and SREBP1 bound to promoters of DGAT1 sequentially or simultaneously. ChIP-qPCR assays at the indicated promoter suggested that BRD9 abundance was impaired with SREBP1 inhibition, whereas SREBP1 binding was not altered with BRD9 knockdown, implicating that SREBP1 could recruit and guide BRD9 binding to the DGAT1 promoter (Fig. 4I). We further investigated the potential for the cooperative binding of BRD9 with RNA polymerase II (RNA Pol II). Previous studies have implicated that BRD9 could directly bind to the SOX4 promoter, and we thus used SOX4 as a positive control in the following assays [28]. The ChIP-qPCR analysis revealed that both Ser5- and Ser2-phosphorylated RNA Pol II CTDs were impaired in response to BRD9 depletion (Fig. 4J). Compared to defective BRD9-Δbromodomain, wild-type BRD9 could completely rescue the enrichment of Ser5- and Ser2-phosphorylated RNA Pol II CTDs at DGAT1 promoters (Fig. 4J). Moreover, ChIP assays further demonstrated that WT BRD9, but not the BRD9-Δbromodomain, was indispensable for sustaining the chromatin accessibility at the DGAT1 or SOX4 promoter (Fig. 4K). Lastly, we intended to explore the BRD9/SREBP1-associated epigenetic factors. Based on the BioGRID platform, we focused on a series of potential BRD9-interacted chromatin remodeling factors, like PHF10, SMARCA2/4, SMARCC2, SMARCD1, or SMARCE1. The siRNA screen and validation confirmed that only SMARCD1 inhibition could suppress the chromatin accessibility of the DGAT1 promoter, thereby down-regulating its mRNA levels (Fig. 4L). Intriguingly, BRD9-KO could completely abrogate the association between SREBP1 and SMARCD1 (Fig. 4M). BRD9 depended on SMARCD1 to increase the chromatin accessibility of the DGAT1 promoter region, and further induced the mRNA levels of DGAT1 (Fig. 4N). Last, we also validated that YAP depended on BRD9 to maintain the chromatin accessibility of the DGAT1 promoter region, and further up-regulate the DGAT1 mRNA levels (Fig. 4O, P). To summarize, our data suggest that BRD9 occupies the DGAT1 promoter and coordinates with SREBP1 to activate its expression in PCa (Fig. 4Q).

### YAP-BRD9 axis controls the LDs formation and ferroptosis sensitivity via upregulating DGAT1 in PCa

**Fig. 5:**
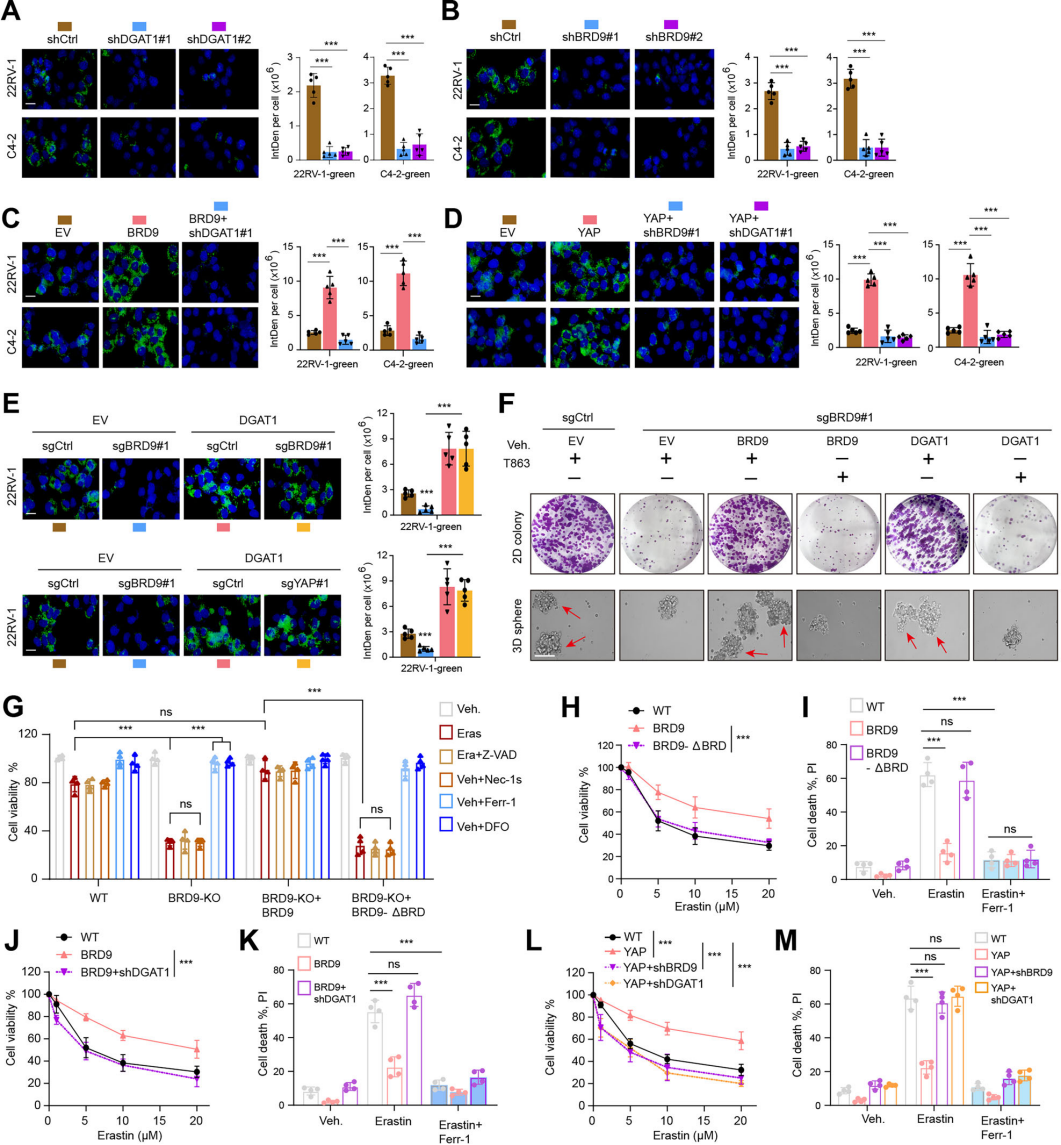
YAP-BRD9 axis drives LDs to mediate ferroptosis resistance via increasing DGAT1 in PCa. **A**, **B** Representative fluorescence imaging (n = 5 graphs) of LDs stained with BODIPY 493/503 (green), and corresponding quantification data in indicated PCa cell lines transduced with shCtrl or DGAT1 shRNA (A), or BRD9 shRNA (B), individually. Nuclei were stained with DAPI (blue). **C** LDs stained with BODIPY 493/503 (green) and quantification data in BRD9-overexpressing cells with or without DGAT1 inhibition. **D** LDs stained with BODIPY 493/503 (green) and quantification data in YAP-overexpressing cells with or without BRD9/DGAT1 inhibition. **E** Representative fluorescence pictures of LDs stained with BODIPY 493/503 (green), and quantification data in BRD9-KO (upper), or YAP-KO (lower) 22RV-1 cells overexpressed with EV or DGAT1. Nuclei were stained with DAPI (blue). **F** Colony formation or sphere-formation assays in BRD9-KO 22RV-1 cells with or without BRD9/DGAT1 restoration. T863 was added to inhibit DGAT1 ability in indicated groups. **G** The column scatter plot revealing the cell viability in 5 µM erastin-treated 22RV-1 cells treated with or without 5 µM Z-VAD, 2 µM Nec-1s, 2 µM Ferr-1 or 100 µM DFO, individually. Ferr-1 or DFO was used to rescue ferroptosis. **H** Cell viability in indicated cells transfected with WT BRD9 or Δbromodomain mutant, assessed after increasing concentrations of erastin treatment for 24 h. **I** Cell death detected (PI) in cells transfected with WT BRD9 or Δbromodomain mutant after treatment with Ferr-1 (2 µM) and erastin (5 µM) for 24 h. **J** Cell viability in BRD9-overexpressing cells with or without DGAT1 inhibition, assessed after increasing doses of erastin treatment for 24 h. **K** Cell death detected (PI) in indicated cells after treatment of Ferr-1 and erastin. **L** Cell viability showing the erastin sensitivity in YAP-overexpressing cells with or without BRD9/DGAT1 inhibition. **M** Quantification of ferroptosis-induced cell death (PI method) in YAP-overexpressing cells with or without BRD9/DGAT1 inhibition. *P < 0.05, **P < 0.01, ***P < 0.001, and ns means no significance. Error bars represent SEM, two-tailed Student’s *t* test.

As reported, DGAT1 is a TG synthase required for the storage of intracellular lipids and may play a role in PCa progression [29]. We thus postulated that BRD9-DGAT1 may contribute to lipid droplet formation in PCa. First, we examined the lipid droplet formation with the Bodipy dye in PCa cells with DGAT1 depletion and observed that DGAT1 knockdown could attenuate the lipid droplet accumulation (Fig. 5A). Next, we also confirmed that BRD9-KD could decrease the lipid droplet formation, which could be completely rescued by BRD9 overexpression (Fig. 5B). In addition, BRD9 depended on DGAT1 to increase lipid droplet formation in PCa cells (Fig. 5C). Consistently, YAP could also enhance the lipid droplet formation, which could be largely abrogated by BRD9 or DGAT1 depletion (Fig. 5D). In contrast, DGAT1 overexpression in BRD9-KD or YAP-KD 22RV-1 cells could largely rescue the effects of YAP or BRD9 depletion (Fig. 5E). However, BRD9-Δbromodomain was defective in driving lipid droplet accumulation (Figure S4A). To validate whether lipid droplet formation could contribute to PCa growth, the T863, an inhibitor of de novo synthesis of fatty acids and subsequent TG storage, was used to treat 22RV-1 and C4-2 cells. As expected, T863 administration in PCa cells significantly impaired the effect of BRD9 overexpression, indicating that lipid droplets are at least partially required for the oncogenic role of BRD9 in PCa growth (Fig. 5F).

Previous studies indicated that DGAT1-dependent lipid droplet formation could sequester excessive polyunsaturated fatty acids (PUFAs) that accumulate in arrested cells in triacylglycerols (TAGs), thus leading to ferroptosis suppression. We next confirmed the potential role of the BRD9-DGAT1 axis in regulating ferroptosis. We observed that treatment with erastin could lead to substantially more cell death in BRD9-KO cells, which could be rescued by WT BRD9, but not the BRD9-Δbromodomain mutant (Fig. 5G). Of note, the erastin-induced cell death could be largely rescued by the ferroptosis inhibitor, ferrostatin or the iron chelator deferoxamine (DFO), but not by the apoptosis inhibitor, Z-VAD-fmk, or the necroptosis inhibitor necrostatin-1s (Fig. 5G). Consistently, BRD9-WT, but not the defective BRD9-Δbromodomain, remarkably attenuated the erastin-induced cell death (Fig. 5H). The erastin-induced cell death could be fully suppressed by ferrostatin (Fig. 5I). In contrast, BRD9 inhibition could significantly enhance the erastin-induced cell death, which could be abrogated by DGAT1 overexpression (Fig. 5J, K). As expected, YAP also relied on BRD9 or DGAT1 to inhibit erastin-induced cell death (Fig. 5L, M). Knocking down BRD9 or DGAT1 coud also decrease the SLC7A11 expressions (Figure S4B). Together, these results suggested that YAP/BRD9 promotes ferroptosis resistance in a DGAT1-dependent manner.

### Targeting BRD9 or DGAT1 with small-molecule inhibitors is an effective strategy for PCa

**Fig. 6:**
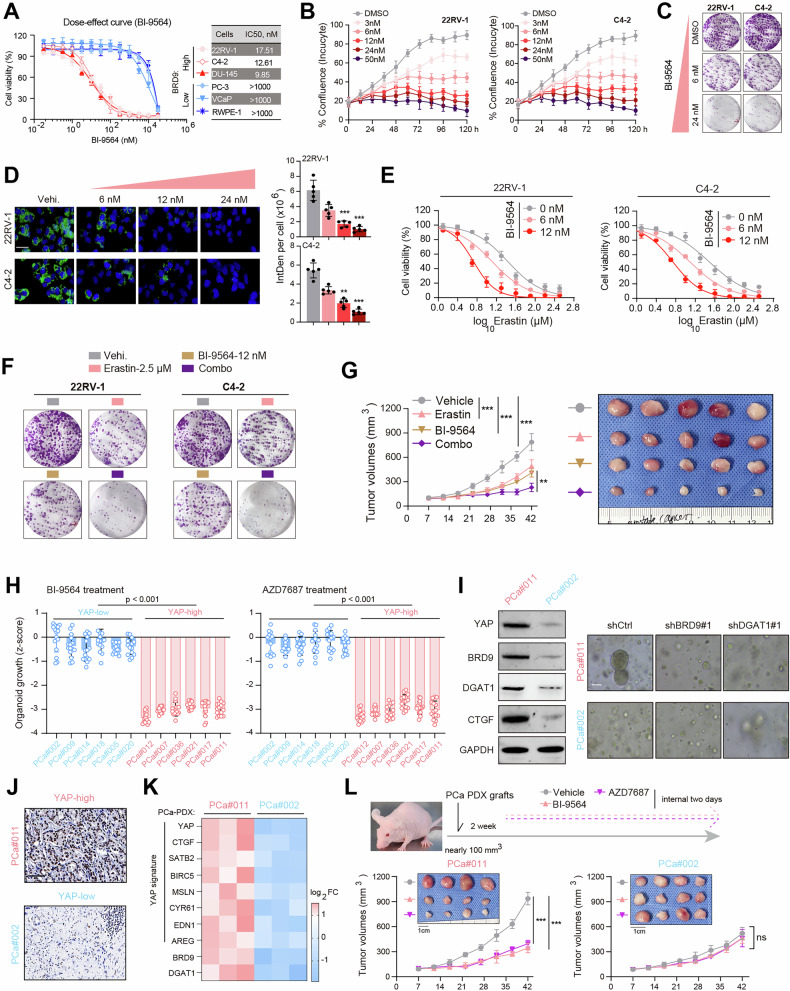
Abrogating BRD9-DGAT1 axis with specific inhibitors for YAP-high PCa. **A** The dose-effect curve showing the IC50 data of BI-9564 in a list of BRD9-high and -low cell lines. **B** Growth curves of 22RV-1 or C4-2 PCa cells upon treatment with increasing concentrations of BI-9564. **C** Colony formation assays showing the growth inhibition of 22RV-1 and C4-2 cells upon treatment of increasing concentrations of BI-9564. **D** Representative LDs IF graphs and statistical data of 22RV-1 and C4-2 cells upon treatment of increasing concentrations of BI-9564. **E** Cell viability curve showing the IC50 data of erastin in 22RV-1 or C4-2 cells treated with indicated doses of BI-9564. **F** Colony formation assays showing the efficacy of erastin, BI-9564, and combination strategy in suppressing 22RV-1 cells. The combination doses for each drug were BI-9564 (6 nM) and erastin (1.5 μM). **G** Representative tumor graph (below) and quantification data (upper) showing the in vivo growth inhibition induced by erastin, BI-9564, and combination strategy (BI-9564: 14 mg/kg IP daily, Erastin: 5 mg/kg). **H** Growth inhibition (Z-score) in PCa organoids grouped by YAP-level under 2 week treatment with indicated compounds targeting BRD9 or DGAT1. **I** Western blot and representative PDOs graphs showing the growth inhibition of YAP-high and YAP-low PCa organoids with or without BRD9-, or DGAT1-KO. The YAP-downstream target, CTGF, is used as the positive control. **J** Representative IHC pictures confirming the YAP-high, or -low expressions in indicated PDXs tissues. **K** Expression heatmap showing the altered mRNA levels of canonical YAP-downstream genes in the indicated PCa-PDXs normalized by the mean value of each signature across all samples. **L** Tumor graphs and corresponding curve data showing the dynamic tumor growth of indicated PCa-PDXs treated with BI-9564 or AZD7687. *P < 0.05, **P < 0.01, ***P < 0.001, and ns means no significance. Error bars represent SEM, two-tailed Student’s *t* test.

Considering that the bromodomain is essential for BRD9 in driving PCa progression, we considered introducing specific inhibitor BI-9564, a cell-permeable BRD9 bromodomain inhibitor [30]. First, the dose-effect curve analysis showed that BRD9-high PCa cells (22RV-1, C4-2, DU-145) were sensitive to BI-9564 treatment with IC50 < 100 nM (Fig. 6A and Figure S5A). However, BRD9-low PCa cells (PC-3, VCaP) or normal prostate epithelium RWPE-1 cells exhibited intrinsic resistance to BI-9564 (IC50 > 10000 nM, Fig. 6A and Figure S5A). BI-9564 could effectively inhibit the cell proliferation, colony formation, and lipid droplet formation of BRD9-high PCa cells (22RV-1, C4-2) in a dose-dependent manner (Fig. 6B–D). In line with previous data, BI-9564 could render PCa cells (22RV-1, C4-2) more sensitive to erastin treatment (Fig. 6E). Combination strategy of BI-9564 and erastin could induce more reduction in cell clony formation numbers than those treated with one drug alone, suggesting the significance of synergistic effects between BI-9564 and erastin (Fig. 6F). Next, we constructed the 22RV-1-derived subcutaneous tumor model, and treated the mice with or without BI-9564. BI-9564 administration failed to induce notable drug toxicity during the observation period, as evidenced by mouse weight or blood routine examination (Figure S5B, C). We then randomly divided these mice into four groups for subsequent drug assays. As indicated by the tumor growth curve, we found that BI-9564 treatment could notably induce tumor suppression (Fig. 6G). Combination of BI-9564 and erastin was shown to be more effective in inhibiting in vivo tumor growth, as compared to either one drug alone (Fig. 6G).

We further put forward the hypothesis that YAP-high PCa might be more sensitive to the BRD9 or DGAT1 inhibitors. First of all, we selected typical YAP targets to reflect its activity, like CTGF, CYR61, BIRC5, as well as AREG [31]. We integrated and named it as the YAP-signature. Waterfall plot indicated that PCa organoids could be categorized into YAP-high and YAP-low groups based on the YAP-signature activity (Figure S5D). Waterfall plots revealed that YAP-high PCa organoids were more sensitive to BI-9564 or AZD7687 (DGAT1 inhibitor) treatment than YAP-low organoids (Fig. 6H). Indeed, YAP-high PCa organoids were more sensitive to BRD9 or DGAT1 depletion compared with YAP-low organoids (Fig. 6I). To further confirm this result, we obtained PCa patient-derived xenografts (PDXs) that show distinct high or low YAP activity (Fig. 6J). We observed that the YAP-signature was positively associated with BRD9-DGAT1 levels (Fig. 6K). Reflected by the quantified tumor volumes, BI-9564 or AZD7687 treatment was more efficient at inhibiting YAP-high PCa-PDX tumors than YAP-low PDX tumors (Fig. 6L). To summarize, we conclude that YAP-high PCa shows vulnerability to the specific BRD9 or DGAT1 inhibitors (Graphical abstract).

## Discussion

Accumulated studies have indicated that DNA methylation, histone acetylation, and chromatin accessibility for transcriptional program play important roles in PCa initiation and progression [32, 33]. Thorough explanation of how epigenetic machinery controls the progression of PCa and the crosstalk between epigenetic and genetic modulators driving PCa may provide a valuable risk prediction and more effective treatment options for patients [34]. Based on functional screen of SWI/SNF subunits, we identified that epigenetic reader BRD9 is essential for growth and progression of PCa. The in vitro assays and murine PCa models confirmed that BRD9 could enhance PCa distal metastasis and self-renewal abilities. The YAP/TEAD4 complex bound the promoters of BRD9 to activate its expressions and stimulated PCa progression. BRD9 interacted with SREBP1 to elevate DGAT1 expressions, therefore increasing fatty acid metabolism and LDs formation. Intriguingly, YAP/BRD9 further mediated ferroptosis resistance of PCa cells in a DGAT1-dependent manner. Lastly, BRD9 specific inhibitor, BI-9564, could efficiently suppress the in vivo growth of YAP-high PCa tumors, and induced the PCa cells sensitivity to ferroptosis inducers. We generated the PCa patient-derived xenografts (PDXs) or organoids and validated that BI-9564 could exhibit synergistic effects with erastin.

Recent researches have implicated that BRD9 could utilize its bromodomain to recruit chromatin modifiers and transcription factors to maintain unique transcriptional programs involved in cell growth, stemness, apoptosis, and drug resistance [28, 35, 36]. In our study, we mainly discovered two layers of DGAT1 activation by BRD9 in tumor cells and ferroptosis resistance: (1) SREBP1 facilitated the BRD9 binding to the DGAT1 promoter and (2) mediated the recruitment of SMARCD1 to open the chromatin accessibility of BRD9-binding regions. In line with previous opinions, the bromodomain was also required for BRD9 to exert its oncogenic functions. Particularly, the BRD9-Δbromodomain truncation mutant failed to increase DGAT1 levels, cholesterol biosynthesis, and modulate PCa ferroptosis sensitivity. The fundamental reason of this phenomenon was that bromodomain is essential for endogenous interactions between BRD9 and SREBP1. ChIP-qPCR assays confirmed that SREBP1 guided BRD9 binding to the promoter of DGAT1, and BRD9 was responsible for transcriptional initiation and activation of SREBP1 via shaping chromatin spatial structure. Aktan Alpsoy et al. previously found that BRD9 interacts with AR and drives the progression of AR-positive PCa [17]. However, our study may propose a new perspective that BRD9 could also regulate AR-independent pathways in PCa, like SREBP1-DGAT1 axis. In addition, the independence and dependence of BRD9 and AR need further validations. For instance, we should demonstrate the regulatory relationship between the SREBP1-DGAT1 axis and AR based on immunoprecipitation and CUT&Tag-sequencing assays. As is well documented, DGAT1 is a vital TG synthase needed for the storage of intracellular lipids. Previous studies indicated the oncogenic roles of DGAT1 in multiple malignancies, especially in PCa. For instance, targeting DGAT1 inhibited PCa cells growth by inducing autophagy flux blockage via oxidative stress [37]. Furthermore, DGAT1 upregulation protects glioblastoma from oxidative damage and sustains lipid homeostasis by enhancing storage of excess FAs [38]. However, the upstream regulators of DGAT1 in various tumors remain largely indefinite. Previous study suggested that JMJD6 controls LDs and kidney cancer by interacting with RBM39 to up-regulate DGAT1 expressions [39]. Similarly, we identified the YAP-BRD9 axis as the novel up-stream signaling of DGAT1 in PCa. We further confirmed that targeting DGAT1-dependent LDs formation could substantially abrogate the BRD9-mediated tumor progression. Lastly, we introduced the BRD9 specific inhibitor, BI-9564, to treat PCa. BI-9564 is a potent, selective and cell-permeable BRD9 bromodomains inhibitor, with IC50 of 75 nM [40]. Expectedly, BI-9564 was only suitable and effective for YAP-high PCa tumors, but not the YAP-low subsets. We considered that PCa exhibits a obvious tumor heterogeneity, and there might be no targetable BRD9 proteins in YAP-low PCa.

As indicated, the YAP and TAZ are the main effectors of the Hippo signal transduction pathway that is involved in multiple layered events in tumorigenesis [41, 42]. The role of YAP/TAZ in cancer development is essential in a context-dependent manner. In different tumor subtypes, YAP/TEAD might play different roles in promoting or suppressing PCa growth. For instance, ATXN3 stabilized YAP protein via inhibiting K48-specific poly-ubiquitination process on YAP proteins, thereby enhancing the progression of PCa [43]. In contrast, YAP impedes AR^+^ PCa growth by antagonizing TEAD-mediated AR signaling in PCa [44]. In our study, we confirmed that YAP/TEAD4 complex could potentiate PCa growth or metastasis partially depending on BRD9. From this point, we uncovered the other downstream signaling of oncogenic YAP/TEAD4 in PCa, and further explained the up-stream regulator of BRD9. Additionally, Qiang Pan et al. suggested that oncogenic activity of YAP relies largely on ZMYND8 to enhance intracellular de novo cholesterol biogenesis via modulating the SREBP2 transcriptional activity [31]. Currently, no studies have yet elucidated the relationship between YAP signal and fatty acid metabolism. Our work confirmed the specific role of BRD9-SREBP1 epigenetic complex in linking the enhanced LDs and YAP activation in PCa. Therefore, we recognized that the dependence of YAP on metabolic pathways may vary in different tumors, owing to different transcriptional regulational programme. Moreover, we confirmed the positive relationships between YAP signal and ferroptosis in PCa, in line with other studies. For instance, CYLD regulates cell ferroptosis through Hippo/YAP signaling in PCa progression [45]. Xian Fu et al. found that YAP1 inhibits RSL3-induced castration-resistant PCa cell ferroptosis by driving glutamine uptake and metabolism to GSH [46]. In our study, we explained that YAP-BRD9 promoted the LDs formation to suppress ferroptosis in PCa. Similar research suggested that the (DGAT)-dependent LDs formation could sequester excessive polyunsaturated fatty acids (PUFAs) that accumulate in tumor cells in triacylglycerols (TAGs), resulting in ferroptosis suppression [47]. Chromatin remodeling and transcription factors play both transcription-dependent and -independent roles in ferroptosis. These epigenetic regulators also regulate other types of cell death, depending on their downstream target genes and their binding partners. For instance, JMJD6 hijacks ATF4-dependent glutathione metabolism to confer ferroptosis resistance in SPOP-Mutated prostate cancer [48]. Moreover, VSTM2L protects prostate cancer cells against ferroptosis via inhibiting VDAC1 oligomerization and maintaining mitochondria homeostasis [49]. Therefore, the ferroptosis sensitivity of prostate cancer cells can be effectively regulated by manipulating epigenetic mechanisms, thereby achieving the goal of clinical translational therapy. Based on this finding, we proposed a novel epigenetic mechanism of ferroptosis resistance in PCa, and suggested the combination strategy of erastin and targeting YAP-BRD9 axis.

This study still has some limitations that need to be further addressed. First of all, we should collect more PCa samples from multiple hospitals to quantify the cutoff that categorizes the YAP/BRD9-high or -low patients. Secondly, we failed to assess the role of the adaptive immune response in the antitumor efficacy of BRD9 abrogation, because we only utilized the athymic nude to conduct PCa xenograft assays. The immunocompetent hosts or *Brd9-knockout* transgenic mice would be warranted to solve this issue. Last of all, we should evaluate the safety, side effects and specificity of BRD9 inhibitors with erastin in in a large cohort of human PDXs and relevant PCa mouse models.

Overall, our study suggested that YAP-BRD9 acts as an oncogenic axis to enhance PCa proliferation, LDs formation, and erastin resistance via hijacking SREBP1-DGAT1 transcription. BRD9 coordinates with SREBP1 to elevate DGAT1 expressions via epigenetic remodeling. Therefore, BRD9 or DGAT1 inhibition represents an effective strategy to inhibit YAP-high PCa and induce ferroptosis sensitivity.

## Materials and methods

### Cell lines and culture

The 22RV-1, 293 T, and C4-2 PCa cell lines were purchased from the Cell Bank of the Chinese Academy of Sciences (Shanghai, China). All cells were cultured in RPMI 1640 medium (Hyclone, GE Healthcare Life Sciences, Logan, UT, USA) supplemented with 10% FBS (Biological Industries) and 1% penicillin/streptomycin (Beyotime Institute of Biotechnology, Nanjing, China) at 37 °C with 5% CO_2_. All cell lines underwent recent authentication by short tandem repeat (STR) profiling and were routinely screened to confirm the absence of mycoplasma contamination.

### Human PCa samples

The 50 PCa samples and their paired normal tissues were obtained from patients who underwent radical prostatectomy for PCa at the department of Urology, Guangzhou Institute of Cancer Research, the Affiliated Cancer Hospital, Guangzhou Medical University. All patients have signed informed consent forms before this study. All procedures were in accordance with Declaration of Helsinki and the research protocols were approved by the Research Ethics Committees of Guangzhou Medical University.

### SiRNA transfection

C4-2 cells were seeded in 6-well plates to be 60–70% confluent while 22RV-1 cells were approximately 50% confluent at transfection, followed by transfection using the invitrogen^TM^ lipofectamine^TM^ RNAiMAX reagent (8 μl RNAiMAX and 5 μl 20 μM

siRNA were used per well), and media were changed after 12 h transfection. Cells were incubated for >48 h at 37°C then used to perform downstream experiments (immunoblots, cell proliferation assay, and soft agar colony formation assay). Nontargeting siRNA was used as control.

### Lentivirus packaging and infection

To construct lentiviral particles, HEK 293 T cells at 60%-70% confluence in a 100 mm dish were co-transfected with 3 μg target plasmid, 9 μg psPAX2, 9 μg pMD2.G using Lipofectamine™ 3000 (L3000075, Thermo Fisher, Carlsbad, CA, USA) as a gene delivery carrier. Cells were refreshed with 15 ml full medium after incubated at 37 °C for 6 h and lentiviral particle-enriched supernatant was collected 48 h after virus packaging. Using these lentiviral particles to infect C4-2 or 22RV-1 cells and the stable expressing cells were established with 10 μg/ml puromycin for 2 weeks. The shRNA sequences were used to knockdown the expression of YAP, BRD9, DGAT1, or other indicated targets in PCa cells. The sequences for shRNA in the study were shown as the following: shYAP#1 (5’->3’):

F:CCGGGCCACCAAGCTAGATAAAGAACTCGAGTTCTTTATCTAGCTTGGTGGCTTTTTG,

R:AATTCAAAAAGCCACCAAGCTAGATAAAGAACTCGAGTTCTTTATCTAGCTTGGTGGC;

shYAP#2 (5’->3’):

F:CCGGCCCAGTTAAATGTTCACCAATCTCGAGATTGGTGAACATTTAACTGGGTTTTTG,

R:AATTCAAAAACCCAGTTAAATGTTCACCAATCTCGAGATTGGTGAACATTTAACTGGG;

shTEAD4#1 (5’->3’):

F:CCGGGAGACAGAGTATGCTCGCTATCTCGAGATAGCGAGCATACTCTGTCTCTTTTTG,

R:AATTCAAAAAGAGACAGAGTATGCTCGCTATCTCGAGATAGCGAGCATACTCTGTCTC;

shTEAD4#2 (5’->3’):

F:CCGGGAAGAGACGTGTGTGCAGGAACTCGAGTTCCTGCACACACGTCTCTTCTTTTTG,

R:AATTCAAAAAGAAGAGACGTGTGTGCAGGAACTCGAGTTCCTGCACACACGTCTCTTC;

shBRD9#1 (5’->3’):

F:CCGGGAGAGCACACCTATTCAGCAACTCGAGTTGCTGAATAGGTGTGCTCTCTTTTTG,

R:AATTCAAAAAGAGAGCACACCTATTCAGCAACTCGAGTTGCTGAATAGGTGTGCTCTC;

shBRD9#2 (5’->3’):

F:CCGGGCAATGACATACAATAGGCCACTCGAGTGGCCTATTGTATGTCATTGCTTTTTG,

R:AATTCAAAAAGCAATGACATACAATAGGCCACTCGAGTGGCCTATTGTATGTCATTGC;

shSMARCD1#1 (5’->3’):

F:CCGGCCATGAGACAATAGAAACCATCTCGAGATGGTTTCTATTGTCTCATGGTTTTTG,

R:AATTCAAAAACCATGAGACAATAGAAACCATCTCGAGATGGTTTCTATTGTCTCATGG;

shSMARCD1#2 (5’->3’):

F:CCGGCCAGACAACCATCTGGTAGAACTCGAGTTCTACCAGATGGTTGTCTGGTTTTTG,

R:AATTCAAAAACCAGACAACCATCTGGTAGAACTCGAGTTCTACCAGATGGTTGTCTGG;

shSMARCA2#1 (5’->3’):

F:CCGGCGACTCTATCTAACTGGACATCTCGAGATGTCCAGTTAGATAGAGTCGTTTTTG,

R:AATTCAAAAACGACTCTATCTAACTGGACATCTCGAGATGTCCAGTTAGATAGAGTCG;

shSMARCA2#2 (5’->3’):

F:CCGGCCAAACCTGTAGTGAGCGATTCTCGAGAATCGCTCACTACAGGTTTGGTTTTTG,

R:AATTCAAAAACCAAACCTGTAGTGAGCGATTCTCGAGAATCGCTCACTACAGGTTTGG.

### The CRISPR/Cas9 gene knockout in PCa cells

The design of sgRNAs sequence was performed according to the platform of Zhang’s laboratory (http://crispr.mit.edu/). PCa cells were cultured in a 100 mm dish, and a single lentivirus with sgRNA-Cas9 plasmid was transfected. Two days after transfection, the cells were transferred into a 100 mm dish at a ratio of 1:4 and treated with 2 μg/ml purinomycin after 3 days. The KO efficiency was validated by RT-PCR and Western blotting. Cells transfected with sgRNA were selected using purinamycin. The sgRNA sequences were listed as the following: 1. YAP (5’->3’):

sgYAP#1: ACTCTCATCTCGAGAGTGAT

sgYAP#2: ACGGTTCTGCTGTGAGGGCA;

2. TEAD4 (5’->3’):

sgTEAD4#1: ACTACTCTTACCGCATCCAC

sgTEAD4#2: GATGTCCACGGCTTCGAGGT

3. BRD9 (5’->3’):

sgBRD9#1: AAGCTCGGGACAGGATCAAC

sgBRD9#2: AACTTGTAGTACACGGTATC

4. DGAT1 (5’->3’):

sgDGAT1#1: GTTCGTGGGCCGCTTTTTCC

sgDGAT1#2: GTTGAGCTCGTAGCACAAGG

### Generation of overexpression cell lines

One day in advance, cells were separated for infection. When the cell density reached approximately 70%, the culture medium was aspirated, and viral filtrate was added. Twenty-four hours after infection, the culture medium was replaced with fresh medium, and the cells were cultured for another 24 h. Screening was then performed using the optimal concentration of puromycin. The cell samples were divided into the control group, and the YAP or BRD9 overexpression group.

### EdU/CCK-8 proliferation assays

DNA synthesis rate assessment was conducted utilizing Click-iT EDU Imaging Kits (Beyotime, Beijing, China), and experiments were finished with the method provided by the manufacturer. CCK-8 assay: 10 µl CCK-8 solution (Dojindo, Kumamoto, Japan) was added to 96-well plates at the time specified in the manufacturer’s instructions, which were incubated for 2 × 10^3^ cells, and then the absorbance was measured at 450 nm after continued incubation for 2 h at 37 °C. All experiments were repeated three times.

### Colony formation assays

For the colony formation assay, cells were digested and resuspension into single cells, and 1 × 10^5^ cells/well were seeded into 6-well plate. The cells were cultured in complete medium refreshed every three days. After incubated for 14 days, cells were washed with PBS three times, fixed with 4% paraformaldehyde for 20 min and stained with 0.1% crystal violet for 20 min. After an additional washing with PBS three times, the representative colonies were quantified.

### Cell migration and invasion assays

The wound-healing assay was used to validate cell migration ability. Cells were seeded in 6-well plates, and when the cell density was about 90%, a wound was scratched in the center of the well. Then, the cells were cultured in serum-free medium for 36 h. The wound was observed using an inverted microscope (Olympus, Japan) and measured by ImageJ. Transwell assays were performed via transwell chambers and Matrigel. The 1 × 10^5^ PCa cells in 200 µl serum-free medium were added into the upper chamber precoated with Matrigel, and 600 µl medium containing 20% FBS was added into the lower chamber. After incubation for 48 h, invaded cells were stained with 0.1% crystal violet and confirmed by an inverted microscopy.

### Tumour sphere formation

For the sphere formation assays, 2000 cells per well were added in ultralow attachment plates. The 22RV-1 and C4-2 cells were cultured for a period of ten days in DMEM medium supplemented with 4 mg/mL insulin, 1:50 B27, 20 ng/mL EGF, and 20 ng/mL basic FGF. After 1.5 weeks, tumor sphere counting was conducted under a microscope.

### RNA isolation and quantitative real-time PCR

Total RNA was extracted using TRIzol reagent (Thermo Fisher) following the Manufacturer’s protocol. The 1 μg of total RNA was reversely transcribed into cDNA using the Hi-script II Q RT-Super Mix kit (Vazyme). qPCR was performed using Cham-Q Universal SYBR qPCR Master Mix (Vazyme). ACTB was used as an internal control gene. All data were calculated by the ΔΔCt method and performed in triplicates. All primers used in this study were listed as the following: 1. YAP (5’->3’):

F: TAGCCCTGCGTAGCCAGTTA

R: TCATGCTTAGTCCACTGTCTGT;

2. TEAD4 (5’->3’):

F: GGACACTACTCTTACCGCATCC

R: TCAAAGACATAGGCAATGCACA;

3. BRD9 (5’->3’):

F: GGCAAGATGGGCTATCTGAAG

R: GGGAGTAGCTTACTGGAGAGC;

4. SOX2 (5’->3’):

F: TACAGCATGTCCTACTCGCAG

R: GAGGAAGAGGTAACCACAGGG;

5. ACSS2 (5’->3’):

F: TTGGGGCTTTGCACTCCATT

R: AGGCATCTGTAGTGATGAGAAGA;

6. SCD1 (5’->3’):

F: GCCCCTCTACTTGGAAGACGA

R: AAGTGATCCCATACAGGGCTC;

7. ACLY (5’->3’):

F: GGTCTCGTTGGGGTCAACC

R: GAAGGGCTCGATCAGAAAGTTC;

8. FDPS (5’->3’):

F: TGTGACCGGCAAAATTGGC

R: GCCCGTTGCAGACACTGAA;

9. MVK (5’->3’):

F: GGGGATTGCGTCAACAGGT

R: GGGTTCCCGTGAATCATTCTC.

### Proximity ligation assays (PLA)

Briefly, cells were seeded in glass bottom cell culture dish. After 24 h, cells were washed with PBS and fixed with 4% PFA for 15 min at room temperature. Cells were then sequentially washed twice with PBS, permeabilized with 0.5% Triton X-100 for 10 min at room temperature, washed three times with PBS, blocked, incubated with antibodies at 4 °C overnight and processed according to the manufacturer’s instructions. Images were acquired with a Leica THUNDER Imager system. Red spots indicate protein–protein interactions, and blue indicates DAPI-stained nuclei.

### Western blotting assays

Protein lysates were prepared by lysing the cells and run on sodium dodecyl sulfate-polyacrylamide gels for electrophoresis (Bio-Rad). Separated proteins were then transferred to the polyvinylidene fluoride (PVDF) membranes. The membranes were blocked with 5% milk and incubated with the primary antibody in the blocking buffer (overnight at 4 °C) followed by horseradish-peroxidase-conjugated secondary antibodies (Proteintech) for 1 h at room temperature. The blots were developed by performing the enhanced chemiluminescence (ECL) detection reagents on the membranes and the signals were detected by the ECL blotting analysis system (Bio-OI, Guangzhou, China). The band intensity was quantified by ImageJ 2.0.0 (ImageJ, NIH). GAPDH was used as the endogenous loading control. The antibodies were listed as the following: anti-YAP (Abcam, ab205270), anti-BRD9 (Abcam, ab259839), anti-CTGF (Abcam, ab6992), anti-DGAT1 (Abcam, ab181180), anti-SMARCD1 (Abcam, ab245222), anti-SREBP1 (Cell signaling technology, #95879), anti-GAPDH (Cell signaling technology, #2118).

### Chromatin immunoprecipitation (ChIP) assays

Cells were cross-linked with 1% formaldehyde solution, neutralized by 1.25 M glycine, then lysed and sonicated. Then the lysates were incubated at 4 °C overnight with the target-specific antibody YAP (Abcam), BRD9 (Cell Signaling Technology), SREBP1 (Abcam), or H3K4me3 (Cell Signaling Technology), or negative control of normal Rabbit IgG. The immunoprecipitated complexes were precleared with ChIP Grade protein A/G Magnetic beads for 2 h. Then DNA was eluted from immunoprecipitated complexes, reverse cross-linked, and purified. High-quality ChIP DNA were used for RT-qPCR as former procedure.

### The CUT&Tag-sequencing and bioinformatic analyses

Cleavage under targets and tagmentation (CUT&Tag) assay was conducted as the standard techniques. Briefly, the cells are bound to concanavalin A-coated magnetic beads, and the cell membrane is permeabilized by digitonin. The enzyme pA-Tn5 transposase precisely binds the DNA sequence near the target protein under the antibody guidance and results in factor-targeted tagmentation. The DNA sequence is tagmented, with adapters added simultaneously at both ends, which PCR enriches to form the sequencing-ready libraries. After the PCR reaction, libraries were purified with the AMPure beads and library quality was assessed on the Agilent Bioanalyzer 2100 system. After sequencing and quality control, the reads were mapped to the reference genome. ChIPseeker was used to retrieve the nearest genes around the peak and annotate the genomic region of the peak. ChIPseeker can confirm peak-related genes.

### Lipid droplet staining and quantification

Briefly, after culturing and indicated treatment, cells were fixed in 4% paraformaldehyde (PFA) for 15 min, washed twice with PBS, then were stained with BODIPY^TM^ 493/503 (1 mM) for 1 h. Cells were then again washed twice with PBS, followed by DAPI or Hoechst 33342 (1:1000) incubation for 5 min, and mounted on slides. Lipid droplets were visualized using a Revolve fluorescence microscope (Echo). Six images were taken in total/group, and lipid droplets levels were quantified by using the ImageJ software (Fiji).

### Xenograft tumor models

For the animal studies, although no formal statistical calculations were used to predetermine sample size, an adequate number of animals was included to capture biologically meaningful effects and accommodate inherent variability. Animals were assigned to different treatment groups through simple randomization. During the execution of the experiments and assessment of outcomes, investigators were aware of group assignments, as blinding was not applicable to the design of this study. Male 5–6-week-old athymic BALB/c nude mice were purchased from the Shanghai Lingchang Technology. The mice were maintained in a special pathogen-free class experimental animal room and kept in an environmentally controlled room (25 °C temperature, 50% humidity and light 12 h/day) with free access to fresh water and a solid pellet diet. Indicated PCa cells (1 × 10^5^) were injected subcutaneously into the left or the right armpit of mice. Subcutaneous tumours were allowed to grow for 20 days. The combination compounds strategy is BI-9564: 14 mg/kg IP daily *plus*. Erastin: 5 mg/kg. Then, the mice were sacrificed using excess carbon dioxide and the subcutaneous tumours were resected and weighed. The animal studies were approved by the Committee on Ethics of Guangzhou Medical University.

### Statistical analyses

The sample size for this study was established to provide sufficient statistical power to detect a predefined effect size, using a significance threshold of 0.05 and a power of 0.8. Power calculations were conducted with G*Power (v3.1.9.7), and effect size estimates were derived from prior publications or preliminary experimental data. Biological samples were excluded only when preparation or data acquisition was unsuccessful; otherwise, no datasets were omitted. For both in vitro and in vivo experiments, samples were assigned to treatment groups through simple randomization. Investigators were aware of group allocation throughout the experiments and outcome assessments, as blinding was not applicable to this study design. All conclusions were drawn strictly from objective quantitative measurements rather than subjective judgment. As indicated in the figure legends, each assay was repeated three or more times. The results are represented as the means ± SD of at least three independent experiments of biological replicates. The two-tailed Student’s *t* test for normally distributed data or the two-tailed Mann-Whitney test for non-normally distributed data were used to test for differences between the two groups. Log-rank test and Kaplan–Meier analysis was used to assess the survival prognosis. *P* < 0.05 was considered statistically significant. GraphPad Prism 9.0. and R software (version 4.2.2) were used to conduct the statistical analysis.

## Supplementary information

Figure S1

Figure S2

Figure S3

Figure S4

Figure S5

Figure S6

Supplementary Figure legends

## Data Availability

The data supporting the findings of this study are available from the corresponding author on reasonable request. The sequencing data generated in this study have been deposited in the NCBI’s Sequence Read Archive (SRA) under the accession number of PRJNA1279341 (Reviewer Link: https://www.ncbi.nlm.nih.gov/sra/?term=PRJNA1279341). The uncropped WB gels were available in Figure S6.
