# Supplementary figures and images for: Aberrant activation of epigenetic BRD9-DGAT1 axis promotes lipid droplets deposition and ferroptosis resistance in YAP-high prostate cancer
Source: Cell Death Dis. 2026 Apr 14;17(1):477. doi: 10.1038/s41419-026-08746-6 (PMC13183962; doi:10.1038/s41419-026-08746-6)

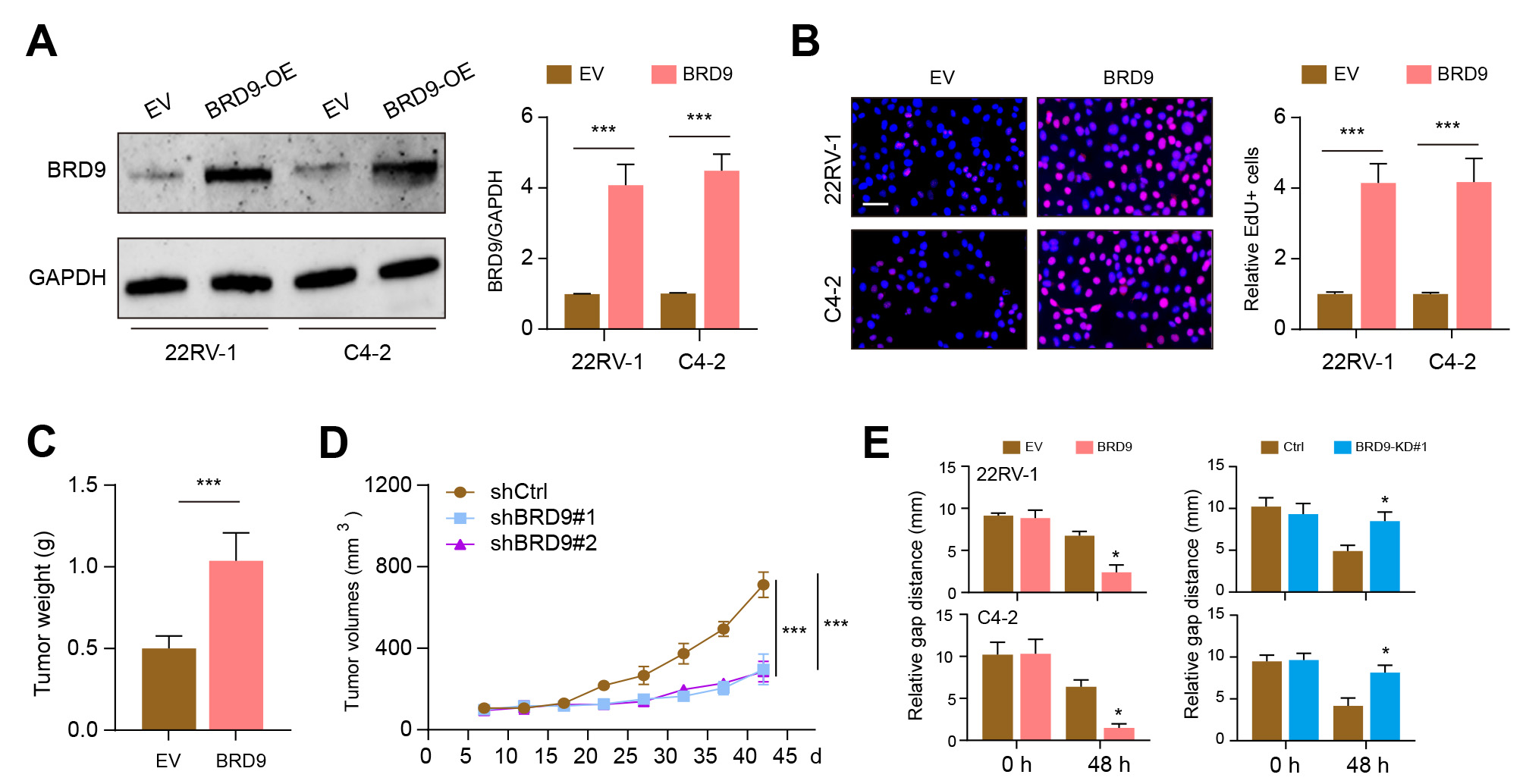

Supplement: Supplementary file 1 — Figure S1 [file 41419_2026_8746_MOESM1_ESM.jpg]

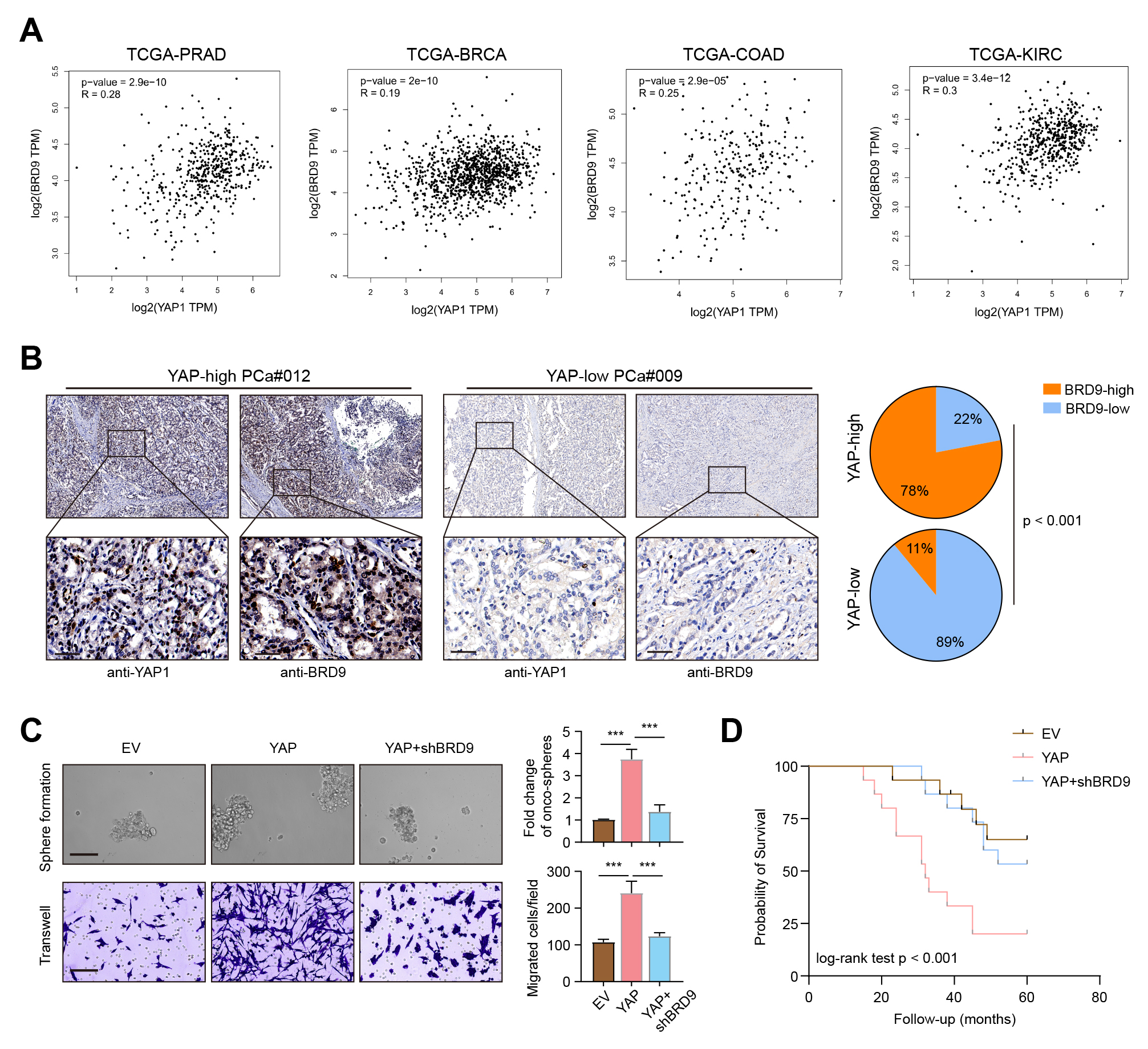

Supplement: Supplementary file 2 — Figure S2 [file 41419_2026_8746_MOESM2_ESM.jpg]

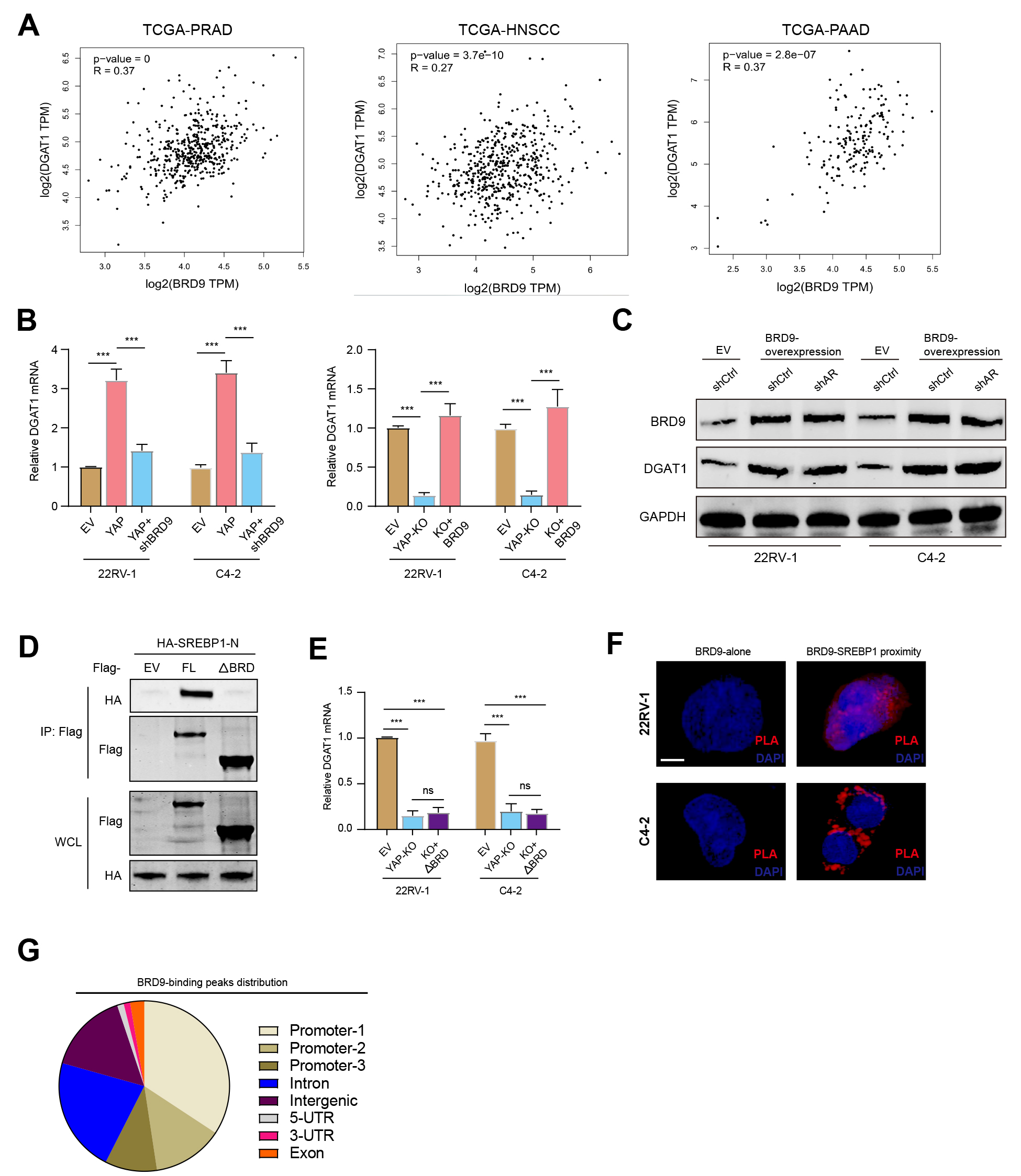

Supplement: Supplementary file 3 — Figure S3 [file 41419_2026_8746_MOESM3_ESM.jpg]

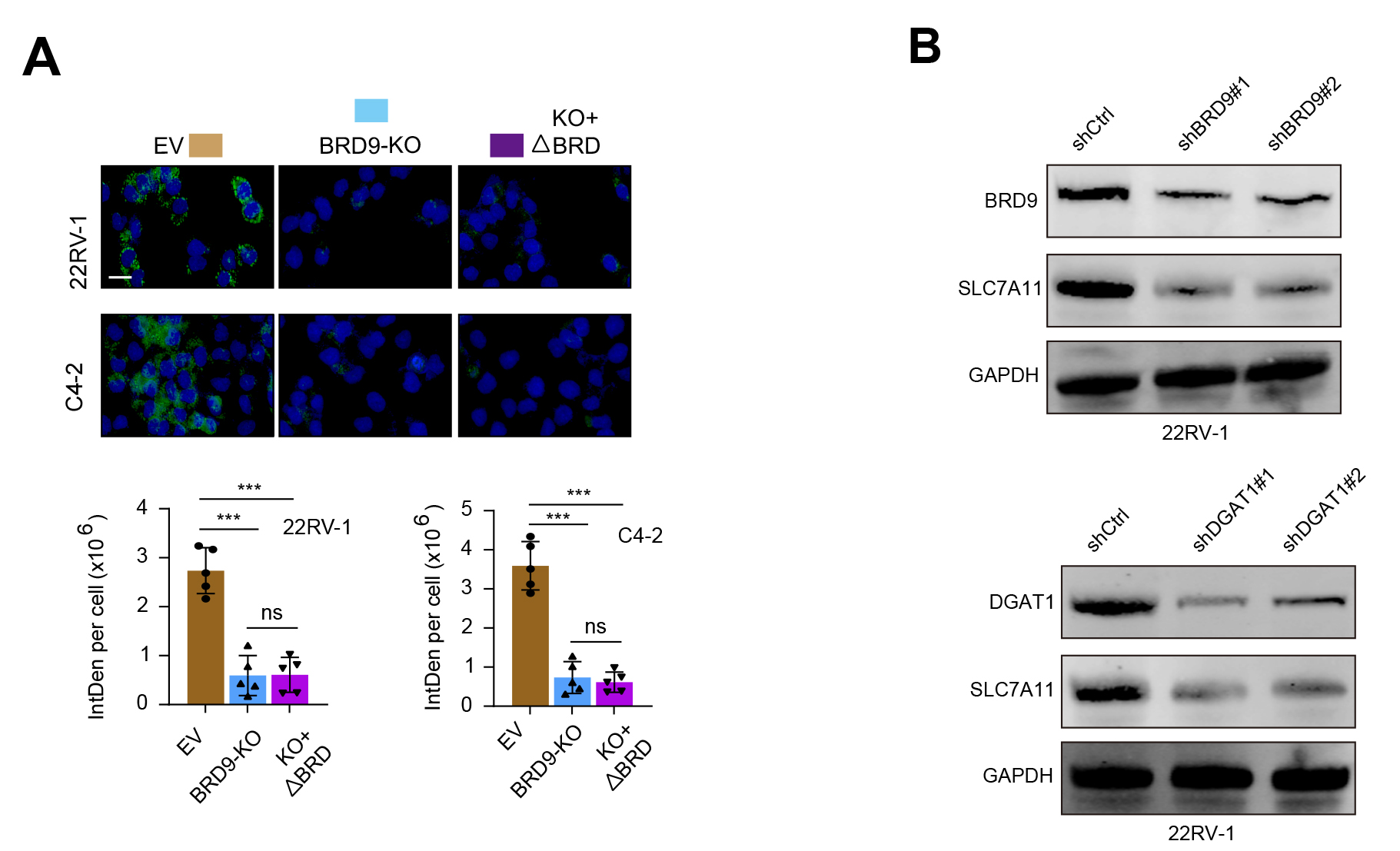

Supplement: Supplementary file 4 — Figure S4 [file 41419_2026_8746_MOESM4_ESM.jpg]

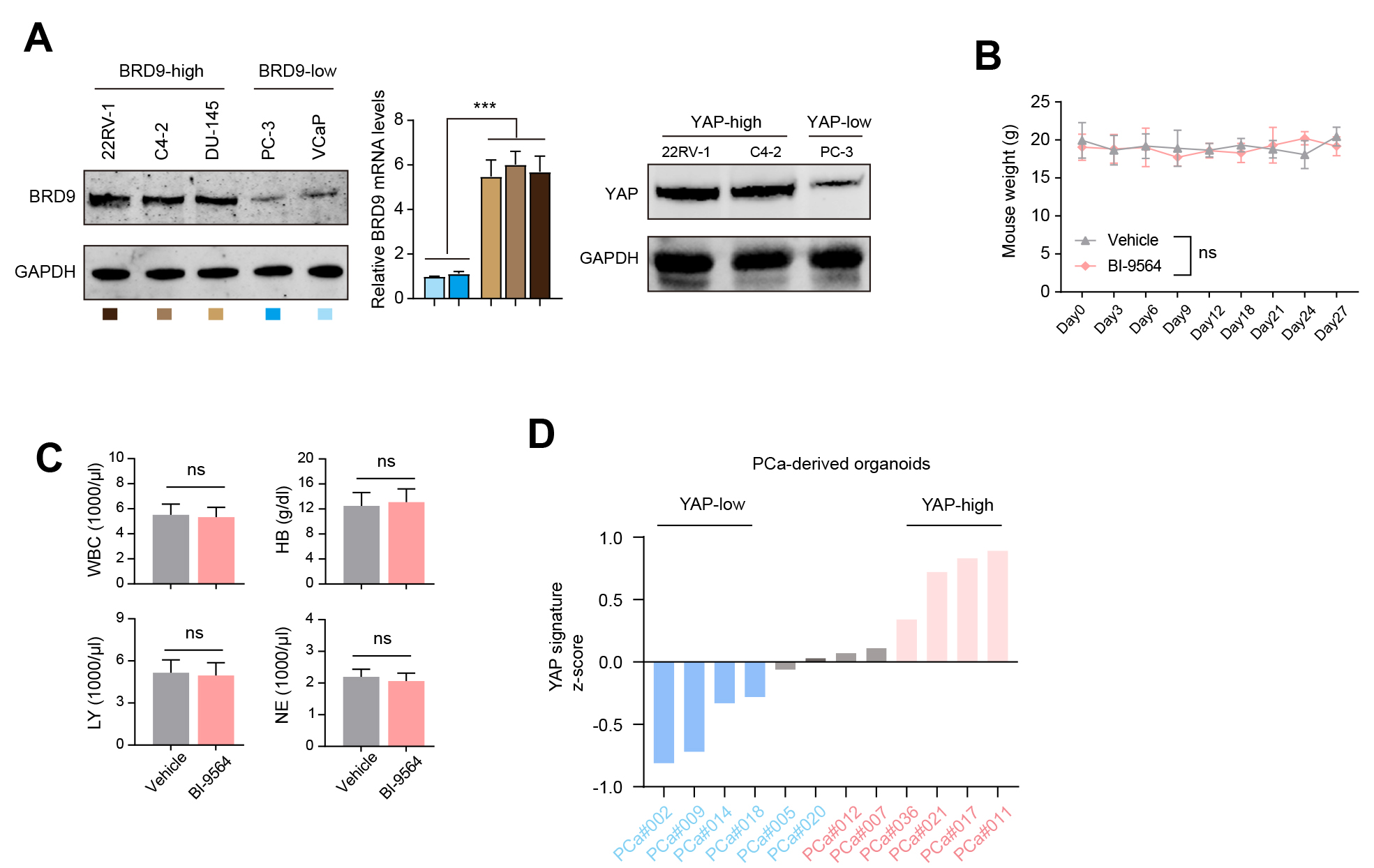

Supplement: Supplementary file 5 — Figure S5 [file 41419_2026_8746_MOESM5_ESM.jpg]
