# Supplementary material for: Aberrant activation of epigenetic BRD9-DGAT1 axis promotes lipid droplets deposition and ferroptosis resistance in YAP-high prostate cancer
Source: Cell Death Dis. 2026 Apr 14;17(1):477. doi: 10.1038/s41419-026-08746-6 (PMC13183962; doi:10.1038/s41419-026-08746-6)

Uncropped WB Gels.

Figure 1E

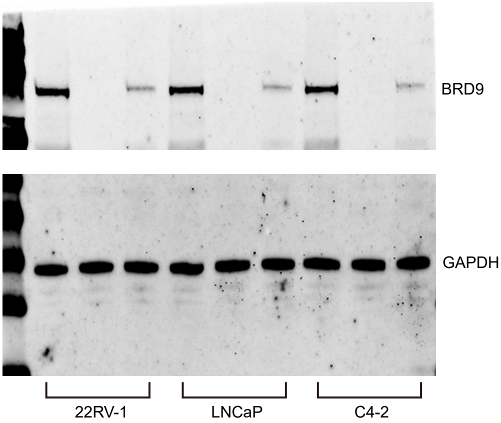

Figure 1G

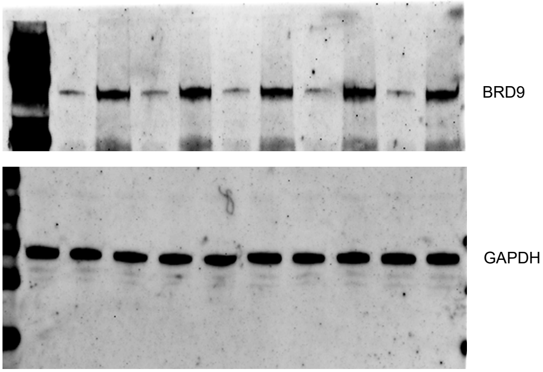

Figure 3B

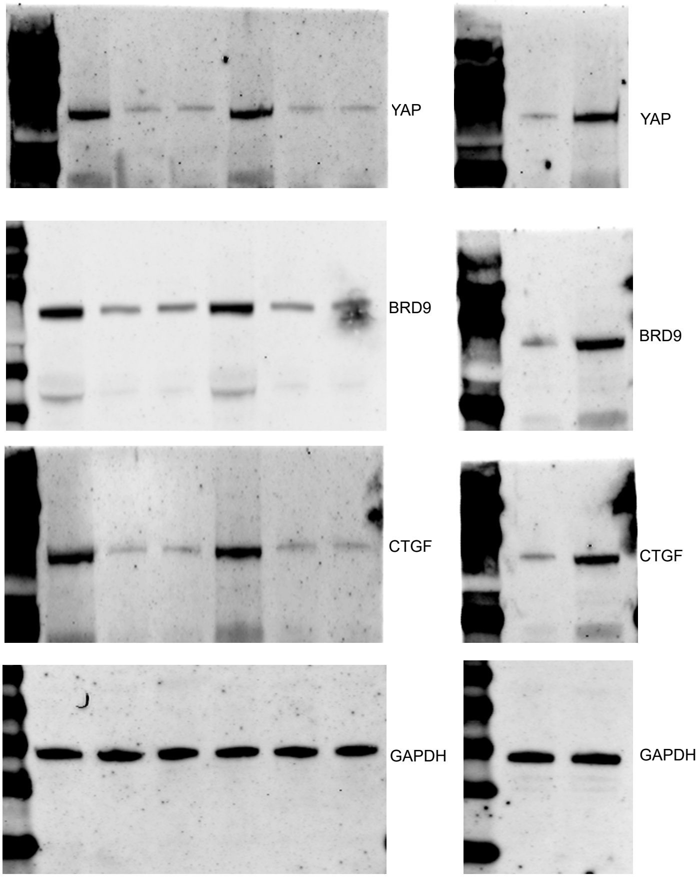

Figure 4F

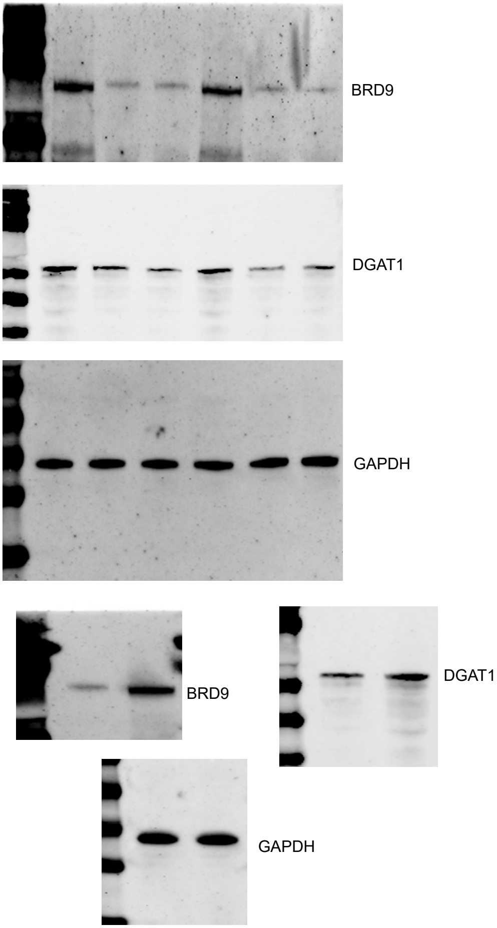

Figure 4G

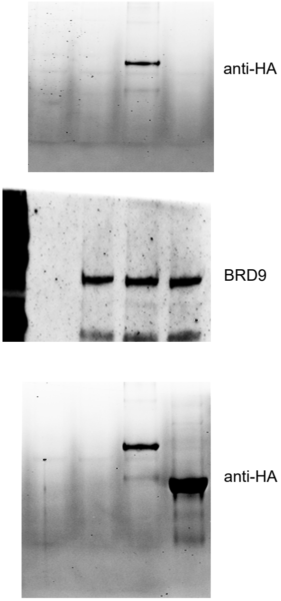

Figure 4M

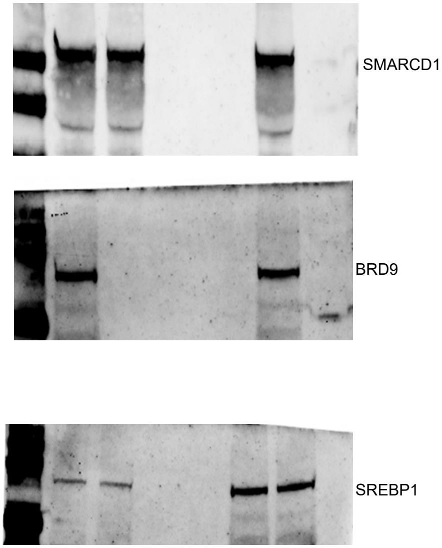

Figure S1A

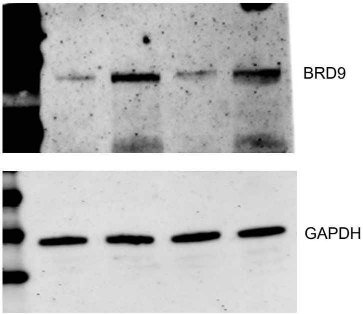

Figure 6I

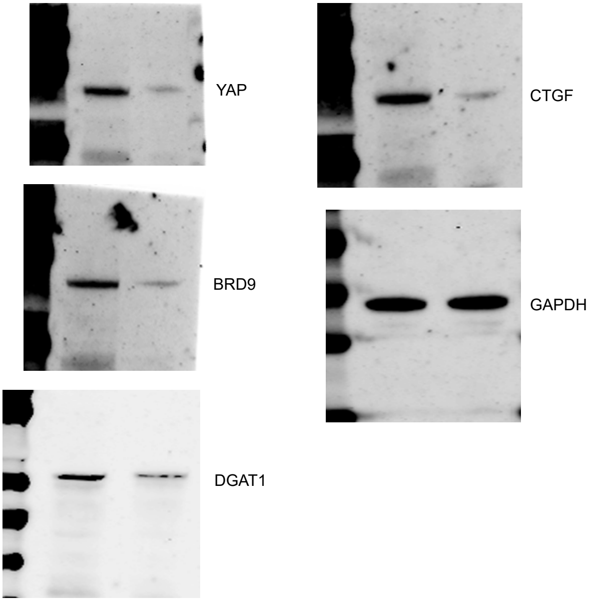

Figure S3C

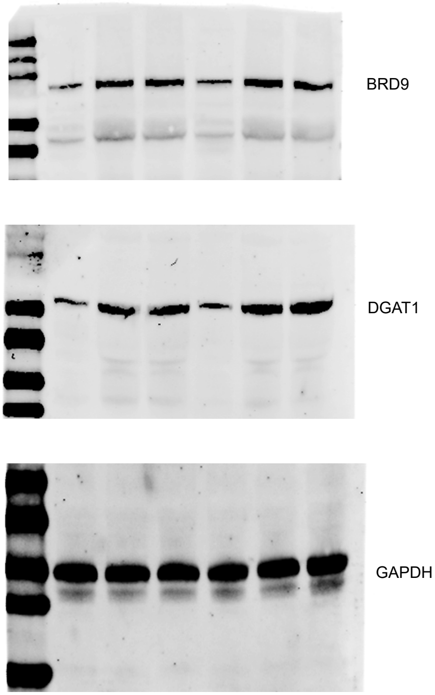

Figure S3D

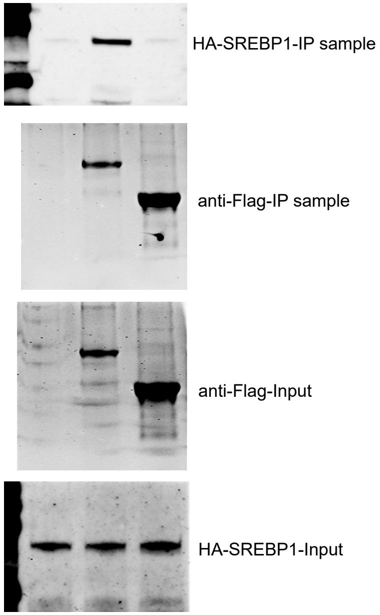

Figure S4B

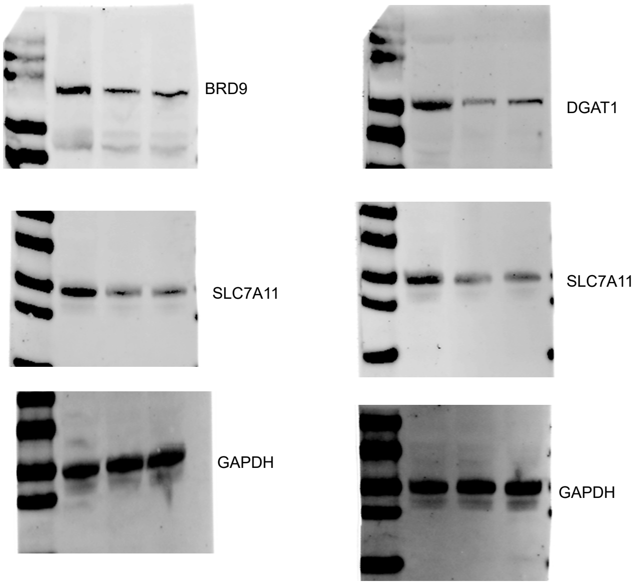

Figure S5A

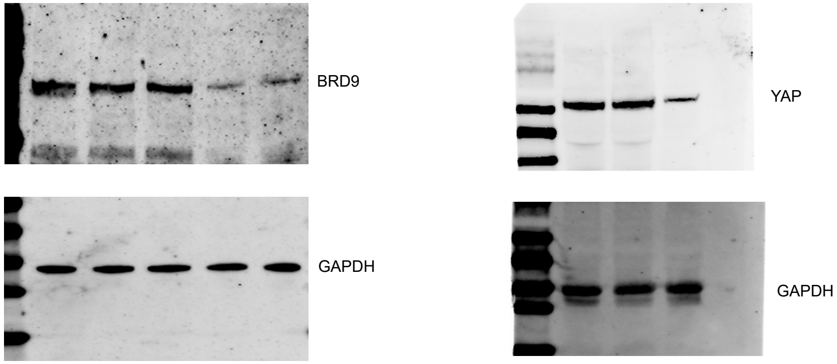

Supplement: Supplementary file 6 — Figure S6 [file 41419_2026_8746_MOESM6_ESM.pdf]
