# Supplementary material for: Aberrant activation of epigenetic BRD9-DGAT1 axis promotes lipid droplets deposition and ferroptosis resistance in YAP-high prostate cancer
Source: Cell Death Dis. 2026 Apr 14;17(1):477. doi: 10.1038/s41419-026-08746-6 (PMC13183962; doi:10.1038/s41419-026-08746-6)
Supplement: Supplementary file 7 — Supplementary Figure legends [file 41419_2026_8746_MOESM7_ESM.docx]

**Supplementary Figure Legends**

**Figure S1 BRD9 accelerates PCa growth, and metastasis of PCa cells. (A)** Western blot assays and RT-qPCR (right) showing the BRD9 expressions in control and BRD9-overexpressing 22RV-1 and C4-2 cells. **(B)** Representative EdU immunofluorescence graphs revealing the growth rates of 22RV-1 or C4-2 cells with or without enforced expressions of BRD9. **(C)** Quantification data of tumor weight in mice from the indicated groups. **(D)** Quantification and comparison of tumor growth curve in tumors derived from shCtrl or shBRD9 groups. **(E)** Quantification and comparison of gap distance between scratch showing the migration abilities of PCa cells. **P* < 0.05, ***P* < 0.01, ****P* < 0.001, and ns means no significance. Error bars represent SEM, two-tailed Student’s t-test.

**Figure S2 YAP positively regulates BRD9 levels in PCa. (A)** Pearson’s correlation analysis between YAP and BRD9 in multiple tumor types based on TCGA data. **(B)** The immunohistochemistry images with anti-YAP or BRD9 showing the expression relationships between them in PCa samples. **(C)** Tumor sphere formation and Transwell assays showing the self-renewal and migration abilities of YAP-sustained PCa cells with or without BRD9 shRNAs. **(D)** Kaplan-Meier analysis showing the prognostic differences in indicated mice. **P* < 0.05, ***P* < 0.01, ****P* < 0.001, and ns means no significance. Error bars represent SEM, two-tailed Student’s t-test.

**Figure S3 YAP-BRD9 enhances LDs formation in PCa. (A)** Pearson’s correlation analysis between BRD9 and DGAT1 in indicated tumor types based on TCGA data. **(B)** The RT-qPCR analysis showing the altered DGAT1 mRNA levels in indicated PCa cells. **(C)** Western blotting assays showing the DGAT1 levels in BRD9-overexpressing PCa cells with or without AR knockdown. **(D)** Western blotting assays detecting the interactions between SREBP1-N with WT BRD9 or BRD9-Δbromodomain mutant in 293 T cells. **(E)** RT-qPCR analysis showing the DGAT1 mRNA levels in YAP-KO C4-2 or 22RV-1 cells with overexpression of BRD9-Δbromodomain. **(F) PLA assays showing the interactions between BRD9 and SREBP1 proteins.** **(G)** The pie chart showing the distribution of BRD9-binding regions based on CUT&Tag-sequencing. **P* < 0.05, ***P* < 0.01, ****P* < 0.001, and ns means no significance. Error bars represent SEM, two-tailed Student’s t-test.

**Figure S4 BRD9-DGAT1 axis regulates the ferroptosis sensitivity of PCa cells. (A)** The immunofluorescence images of LDs showing the cholesterol biosynthesis capacity in BRD9-KO cells with or without overexpression of BRD9-Δbromodomain. **(B)** Western blotting assays showing the SLC7A11 levels in BRD9-deficient PCa cells. **P* < 0.05, ***P* < 0.01, ****P* < 0.001, and ns means no significance. Error bars represent SEM, two-tailed Student’s t-test.

**Figure S5 Targeting BRD9 to induce ferroptosis in PCa. (A)** Western blot assays and RT-qPCR analysis showing the YAP/BRD9 levels in the indicated cell lines. **(B)** Quantification and comparison of mouse weight in mice treated with vehicle or BI-9564, individually. **(C)** Quantitative analysis and comparison of mouse blood parameters. **(D)** Waterfall plot revealing PCa organoids divided into YAP-low and YAP-high based on the z-score derived from mRNA expressions of YAP-regulated signature. **P* < 0.05, ***P* < 0.01, ****P* < 0.001, and ns means no significance. Error bars represent SEM, two-tailed Student’s t-test.

**Figure S6 Summary of uncropped western blot graphs in this study.**
